# Failure of phylogeny inferred from multilocus sequence typing to represent bacterial phylogeny

**DOI:** 10.1038/s41598-017-04707-4

**Published:** 2017-07-03

**Authors:** Alan K. L. Tsang, Hwei Huih Lee, Siu-Ming Yiu, Susanna K. P. Lau, Patrick C. Y. Woo

**Affiliations:** 1 Department of Microbiology, The University of Hong Kong, Pok Fu Lam, Hong Kong; 2 Department of Computer Science, The University of Hong Kong, Pok Fu Lam, Hong Kong; 3 State Key Laboratory of Emerging Infectious Diseases, The University of Hong Kong, Pok Fu Lam, Hong Kong; 4 Research Centre of Infection and Immunology, The University of Hong Kong, Pok Fu Lam, Hong Kong; 5 Carol Yu Centre for Infection, The University of Hong Kong, Pok Fu Lam, Hong Kong; 6 Collaborative Innovation Center for Diagnosis and Treatment of Infectious Diseases, The University of Hong Kong, Pok Fu Lam, Hong Kong

## Abstract

Although multilocus sequence typing (MLST) is highly discriminatory and useful for outbreak investigations and epidemiological surveillance, it has always been controversial whether clustering and phylogeny inferred from the MLST gene loci can represent the real phylogeny of bacterial strains. In this study, we compare the phylogenetic trees constructed using three approaches, (1) concatenated blocks of homologous sequence shared between the bacterial genomes, (2) genome single-nucleotide polymorphisms (SNP) profile and (3) concatenated nucleotide sequences of gene loci in the corresponding MLST schemes, for 10 bacterial species with >30 complete genome sequences available. Major differences in strain clustering at more than one position were observed between the phylogeny inferred using genome/SNP data and MLST for all 10 bacterial species. Shimodaira-Hasegawa test revealed significant difference between the topologies of the genome and MLST trees for nine of the 10 bacterial species, and significant difference between the topologies of the SNP and MLST trees were present for all 10 bacterial species. Matching Clusters and R-F Clusters metrics showed that the distances between the genome/SNP and MLST trees were larger than those between the SNP and genome trees. Phylogeny inferred from MLST failed to represent genome phylogeny with the same bacterial species.

## Introduction

Since the invention of multilocus sequence typing (MLST) in 1998, this technique has been confirmed to be highly reproducible, objective and discriminatory for molecular typing of bacteria, and can be performed easily by different laboratories for typing of strains collected in different localities^1^. MLST involves amplification and sequencing of multiple, usually seven, gene loci. In the past 15 years, MLST has been used widely for typing of bacteria^2–7^. At the moment, MLST schemes are available for more than 110 bacteria. Recently, we have developed an MLST scheme for *Laribacter hongkongensis*, a novel bacterium associated with fish-borne gastroenteritis and traveler’s diarrhea, which was also achieved using seven gene loci^8, 9^. eBURST and minimum spanning trees are commonly used to analyze MLST data for typing or cluster analysis. Despite its high discriminatory power for typing bacteria, it has been controversial whether the phylogenetic tree constructed using the sequences of the gene loci can represent the microevolution process of the bacterial strains undergoing typing, although many studies have used MLST data for further phylogenetic analysis based on the concatenation of the MLST genes^10–14^.

Complete genome sequencing of bacteria has not only revolutionized our understanding on multiple aspects of bacterial genetics and genomics and the phylogenetic relationships among bacteria at the species and intraspecies levels, but the availability of the genome sequences has also given us ample opportunities to solve problems that we have never been able to solve in the past. At the time of writing, more than 6,600 complete genome sequences of more than 1,900 bacterial species are available (https://www.ncbi.nlm.nih.gov/genome/browse/). Recently, we have also published the complete genome sequence of *L*. *hongkongensis* and have used genome sequencing for typing an emerging bacterium, *Elizabethkingia anopheles* ^15, 16^. During the process of constructing the MLST scheme and performing complete genome sequencing of *L*. *hongkongensis*, we were also inspired to answer the question of whether MLST phylogeny can represent the microevolution process of the genomes which can best be depicted by genome phylogeny. In this study, we used various methods to compare the phylogenetic trees constructed using three approaches, (1) concatenated blocks of homologous sequence shared between the bacterial genomes, (2) genome single-nucleotide polymorphisms (SNP) profile and (3) concatenated nucleotide sequences of the gene loci in the corresponding MLST schemes, for 10 bacterial species with more than 30 complete genome sequences available.

## Materials and Methods

### Bacterial genomes

All bacterial species with more than 30 complete genome sequences available by August 1 2015 were included in the analysis. The genome sequences were obtained from the National Center for Biotechnology Information database (Supplementary Table S1) and were further processed for phylogenetic tree construction using the following three methods. Only chromosomes I for *Burkholderia pseudomallei* were used for analysis because all MLST loci for *B*. *pseudomallei* were located on this chromosome.

### Construction of genome phylogenetic tree

The downloaded genomes were aligned with the multiple genome alignment tool Mugsy by using the “-distance 1000” and “-minlength 100” options^17^. The Multiple Alignment Format blocks were concatenated and transformed in FASTA file format using the script available in Galaxy^18–20^. The resulting core alignment was filtered using Gblocks version 0.91b with the minimum length of a block set at 100 (b4 = 100) by removing poorly aligned positions and divergent regions^21^. An approximately maximum likelihood tree was built using FastTree 2, applying the generalized time-reversible model^22^. Outgroups listed in Table 1 were used for rooting the phylogenetic trees. Phylogenetic trees were visualized with MEGA6^23^.

**Table 1:** Information of outgroups and models used to construct maximum likelihood phylogenetic trees for each bacterial species in this study.

Bacteria	Outgroup (GenBank accession no.)	Substitution models for MLST trees^a^
*Burkholderia pseudomallei*	*Burkholderia thailandensis* E254 (CP004381)	TIM1 + I + G
*Campylobacter jejuni*	*Campylobacter coli* 15-537360 (CP006702)	TIM1 + I + G
*Chlamydia trachomatis*	*Chlamydia muridarum* Nigg (AE002160)	TIM3 + I + G
*Escherichia coli* 1	*Escherichia fergusonii* ATCC 35469 (CU928158)	TIM3 + I + G
*Escherichia coli* 2	*Escherichia fergusonii* ATCC 35469 (CU928158)	GTR + I + G
*Helicobacter pylori*	*Helicobacter acinonychis* str. Sheeba (AM260522)	GTR + I + G
	*Helicobacter pylori* SouthAfrica20 (CP006691)	
	*Helicobacter pylori* SouthAfrica7 (CP002336)	
*Klebsiella pneumoniae*	*Klebsiella variicola* strain DSM 15968 (CP010523)	TIM1 + I + G
*Listeria monocytogenes*	*Listeria innocua* Clip11262 (AL592022)	TIM3 + I + G
*Staphylococcus aureus*	*Staphylococcus capitis* subsp. capitis strain AYP1020 (CP007601)	GTR + I + G
*Salmonella enterica*	*Salmonella bongori* serovar 48:z41:–str. RKS3044 (CP006692)	TrN + I + G
*Streptococcus pyogenes*	*Streptococcus dysgalactiae* subsp. equisimilis 167 (AP012976)	TrN + I + G

^a^G, gamma distributed rate of heterogeneity; I, proportion of invariant sites.

### Construction of SNP phylogenetic tree

Core genome SNPs were identified using the Parsnp program included in Harvest. A reference genome was randomly selected using the parameter ‘-r !’^24^. An approximately maximum likelihood tree was constructed from concatenated SNPs using FastTree 2, applying the generalized time-reversible model^22^. Outgroups were assigned as described above for the genome phylogenetic trees.

### MLST sequence identification for construction of MLST phylogenetic tree

For each bacterial species with more than 30 complete genomes available, the nucleotide sequences of the gene loci used in its MLST scheme for one isolate were downloaded from the PubMLST database (http://pubmlst.org/). Two MLST schemes exist for *Escherichia coli*. The sequences obtained were used as queries in BLASTN searches against the downloaded nucleotide sequence of all the genomes of the species^25^. For all the strains of each bacterial species, the nucleotide sequences of the gene loci used in their MLST scheme were aligned independently with ClustalW2 using default settings^26^. Subsequently, Gblocks version 0.91b with the default options was used to concatenate conserved blocks into a single alignment^21^. Once aligned, the appropriate model of evolution was determined using corrected Akaike’s Information Criteria in jModelTest version 2.1.7^27^. Maximum likelihood phylogenetic trees were inferred by using PhyML version 3.0, based on the concatenated alignment with the selected model listed in Table 1 ^28^. Outgroups were assigned as described above for the genome phylogenetic trees.

### Statistical analysis

Shimodaira-Hasegawa tests were performed to determine the congruence between the topologies of the phylogenetic trees derived from genome/SNP data and MLST gene fragments data^29^. Shimodaira-Hasegawa tests were conducted in PAUP* version 4.0b10 using 10000 RELL bootstrap replicates^30^. The null hypothesis in a Shimodaira-Hasegawa test is that two trees being compared are equally good explanations of the data. A P value of less than 0.05 is considered statistically significant to reject the null hypothesis and indicates that the trees are significantly different from one another.

Furthermore, differences among phylogenetic trees obtained from genome, SNP and MLST gene fragments data were evaluated with the software TreeCmp. In this analysis, two topology metrics, Matching Clusters and R-F Clusters, were utilized. A distance value of 0 indicates that the two trees under evaluation are identical and the value increases when they become more different.

## Results

### Bacterial genomes

A total of 639 genomes of 10 bacterial species with more than 30 complete genome sequences were analyzed (Supplementary Table S1). They included *B*. *pseudomallei* (43 genomes), *Campylobacter jejuni* (31 genomes), *Chlamydia trachomatis* (71 genomes), *Escherichia coli* (112 genomes), *Helicobacter pylori* (71 genomes), *Klebsiella pneumoniae* (36 genomes), *Listeria monocytogenes* (46 genomes), *Staphylococcus aureus* (70 genomes), *Salmonella enterica* (124 genomes) and *Streptococcus pyogenes* (35 genomes). The phylogenetic trees constructed using genomes (chromosome I for *B*. *pseudomallei*) and SNP data were compared to those constructed using the seven gene loci in the corresponding MLST schemes.

### Phylogenies and topology comparisons

For each of the 10 bacterial species, phylogenies were inferred using genome, SNP and MLST data. Major differences in strain clustering at more than one position were observed between the phylogeny inferred using genome/SNP data and MLST for all 10 bacterial species (Figs 1–10), with the most prominent differences observed in *B. pseudomallei*, *K. pneumoniae* and *S. enterica* as described below.

**Figure 1:**
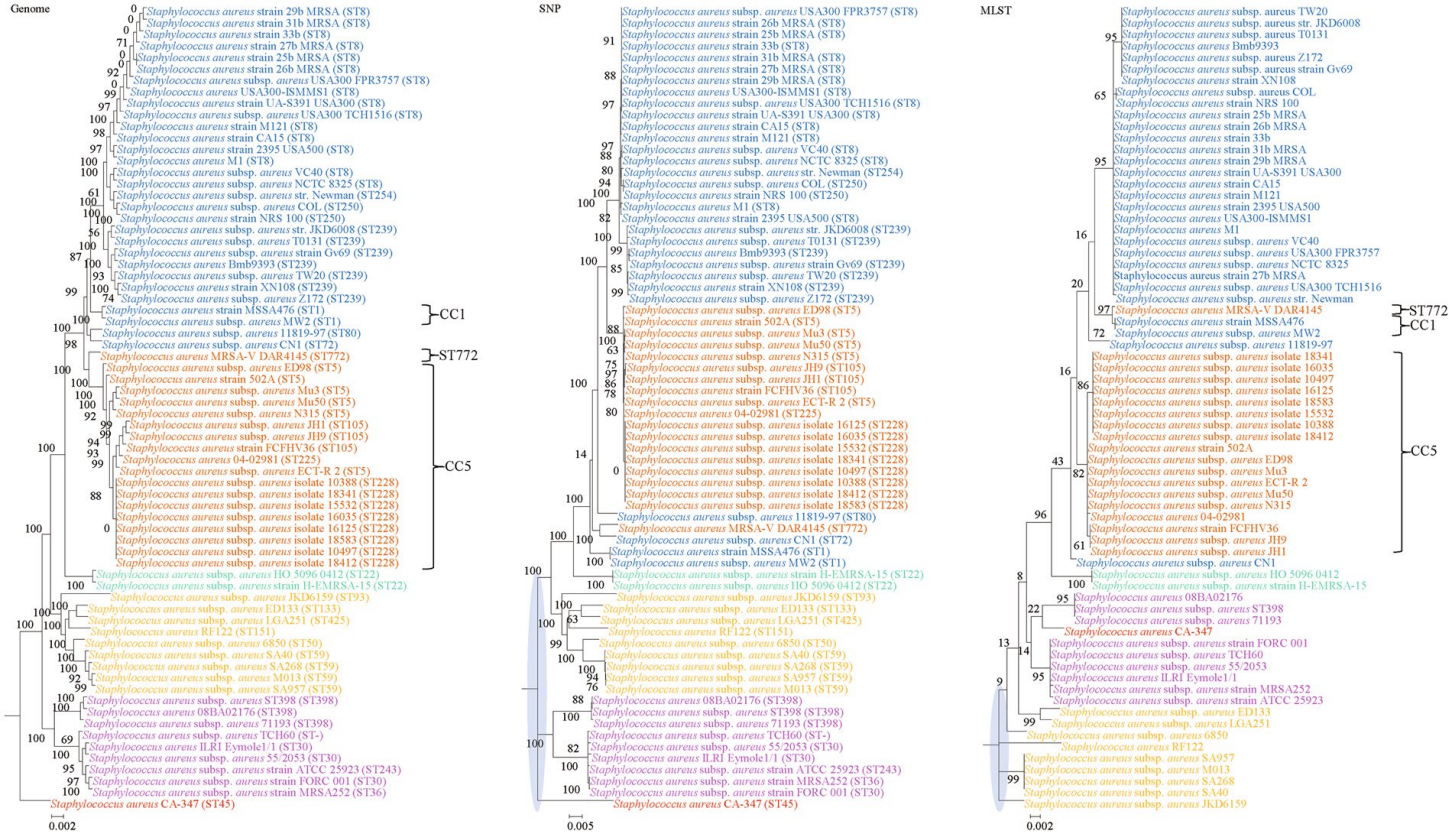
Comparison of phylogenetic trees constructed using genome data (left), SNP data (middle), and MLST data (right) for *Staphylococcus aureus*. Clusters that were manually selected based on the genome trees are illustrated in different colors. The unresolved polytomies are shaded in blue. A new sequence type is represented by a dash (“ST-”).

**Figure 2:**
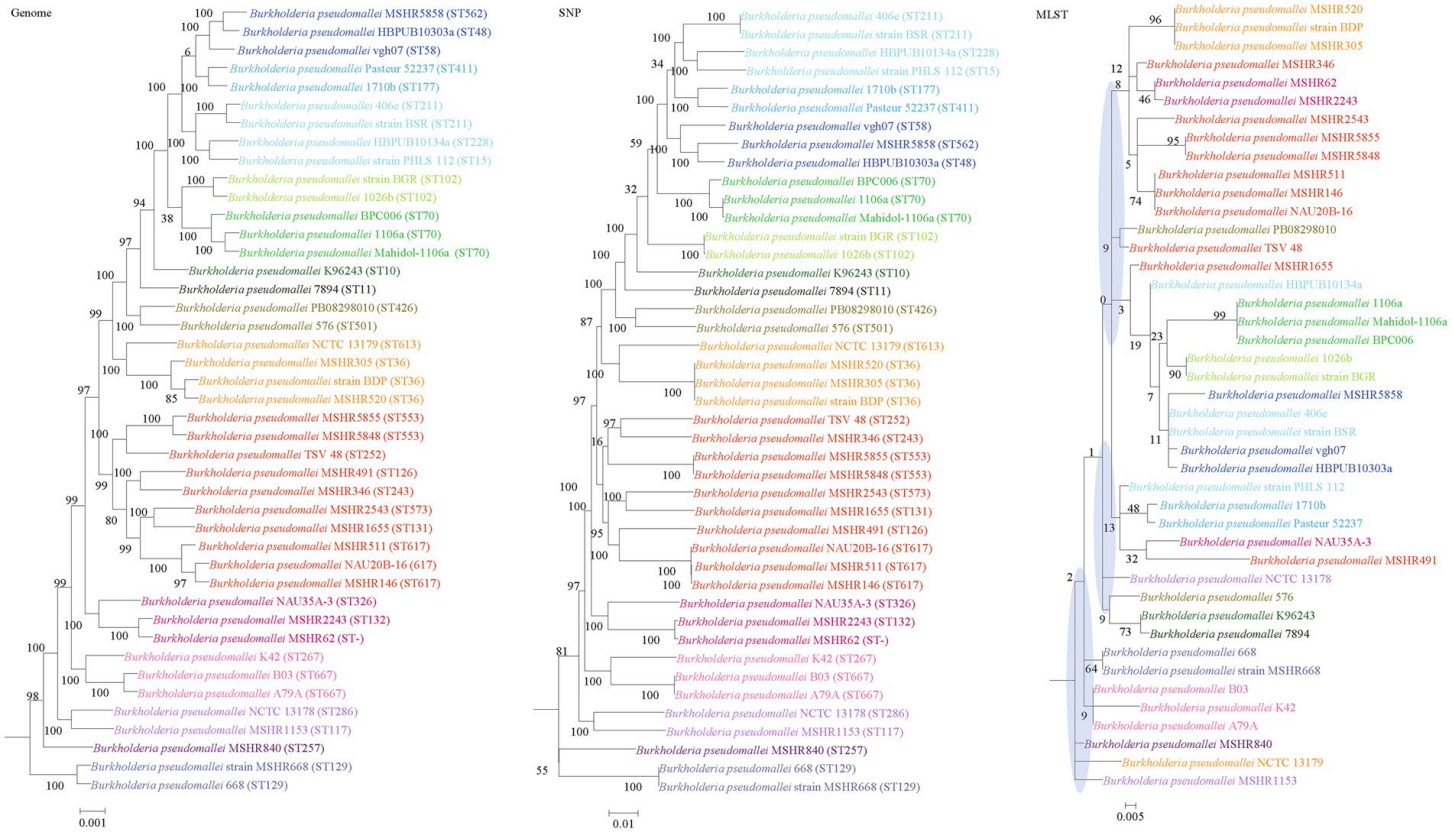
Comparison of phylogenetic trees constructed using genome data (left), SNP data (middle), and MLST data (right) for *Burkholderia pseudomallei* (chromosome I). Clusters that were manually selected based on the genome trees are illustrated in different colors. The unresolved polytomies are shaded in blue. A new sequence type is represented by a dash (“ST-”).

**Figure 3:**
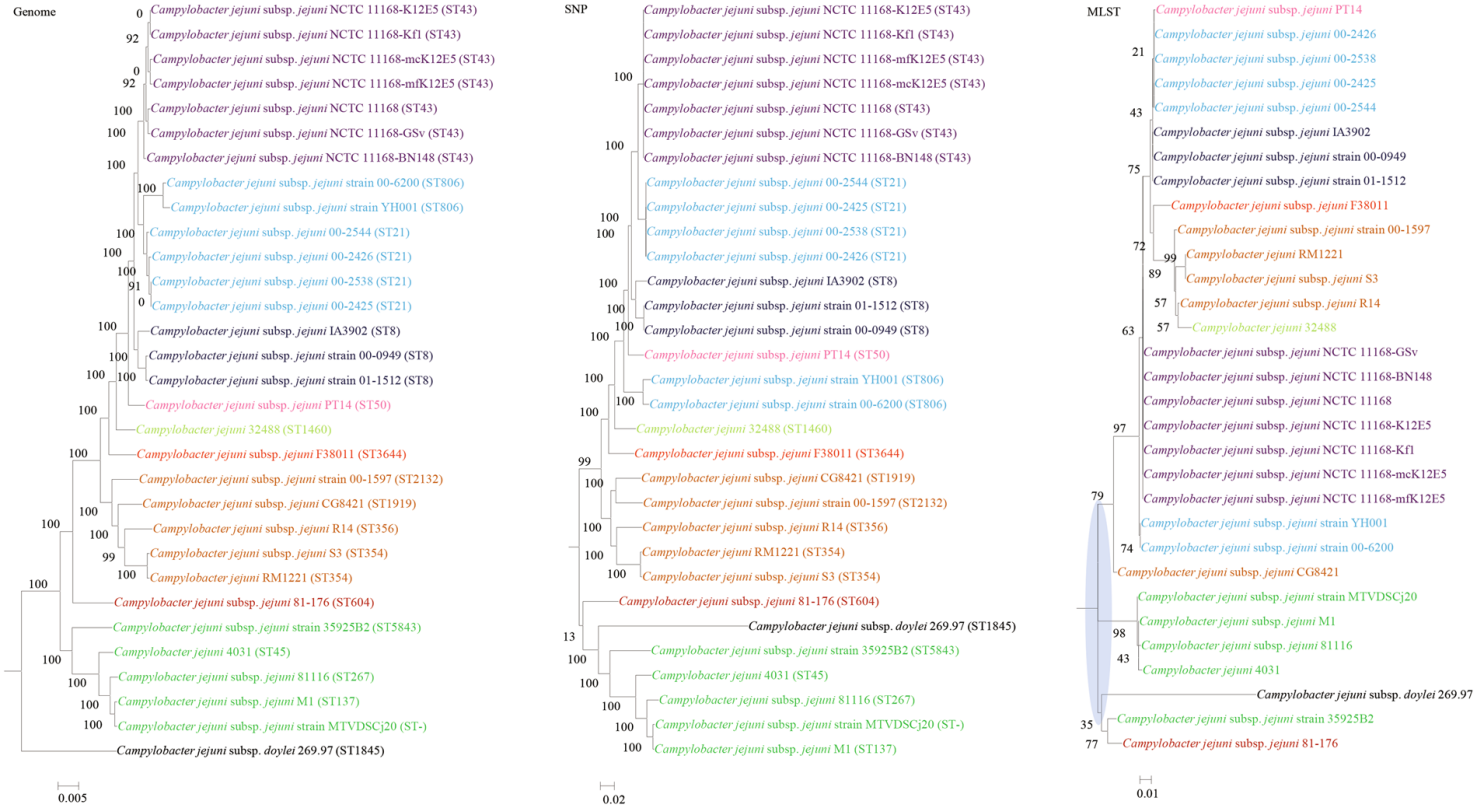
Comparison of phylogenetic trees constructed using genome data (left), SNP data (middle), and MLST data (right) for *Campylobacter jejuni*. Clusters that were manually selected based on the genome trees are illustrated in different colors. The unresolved polytomies are shaded in blue. A new sequence type is represented by a dash (“ST-”).

**Figure 4:**
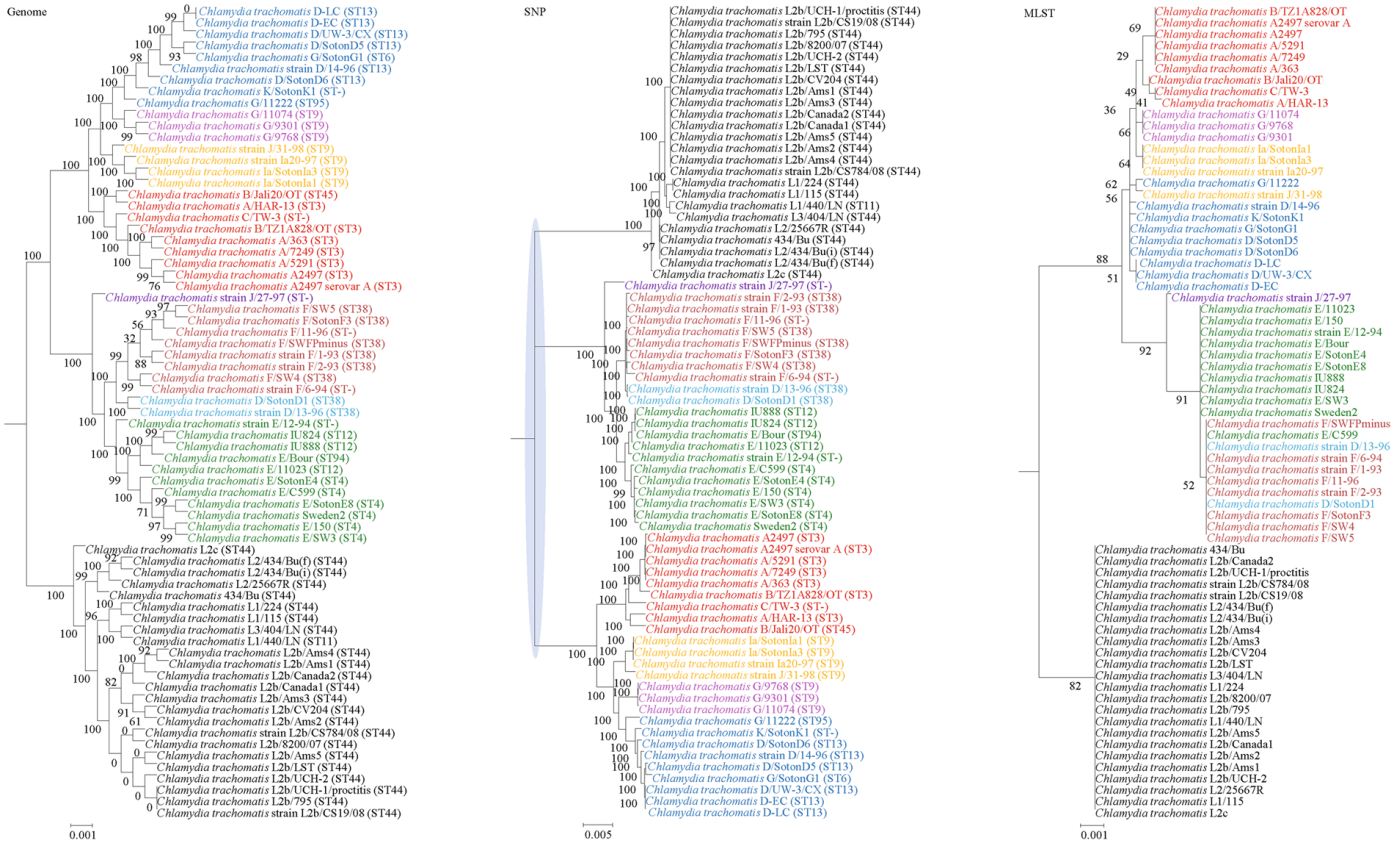
Comparison of phylogenetic trees constructed using genome data (left), SNP data (middle), and MLST data (right) for *Chlamydia trachomatis*. Clusters that were manually selected based on the genome trees are illustrated in different colors. The unresolved polytomies are shaded in blue. A new sequence type is represented by a dash (“ST-”).

**Figure 5:**
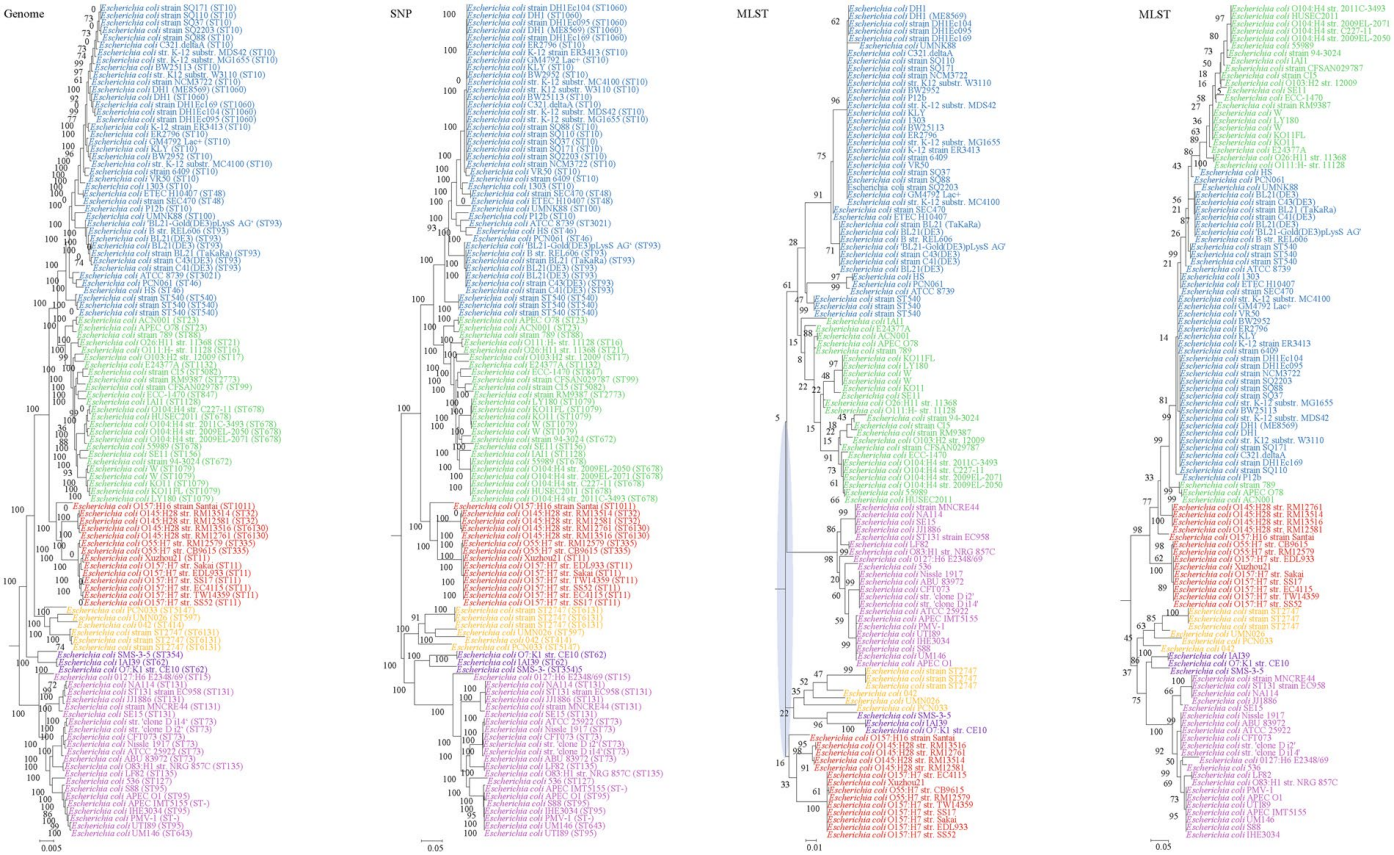
Comparison of phylogenetic trees constructed using genome data (left), SNP data (middle), and MLST data (right) for *Escherichia coli* (two MLST schemes). Clusters that were manually selected based on the genome trees are illustrated in different colors. The unresolved polytomies are shaded in blue. A new sequence type is represented by a dash (“ST-”).

**Figure 6:**
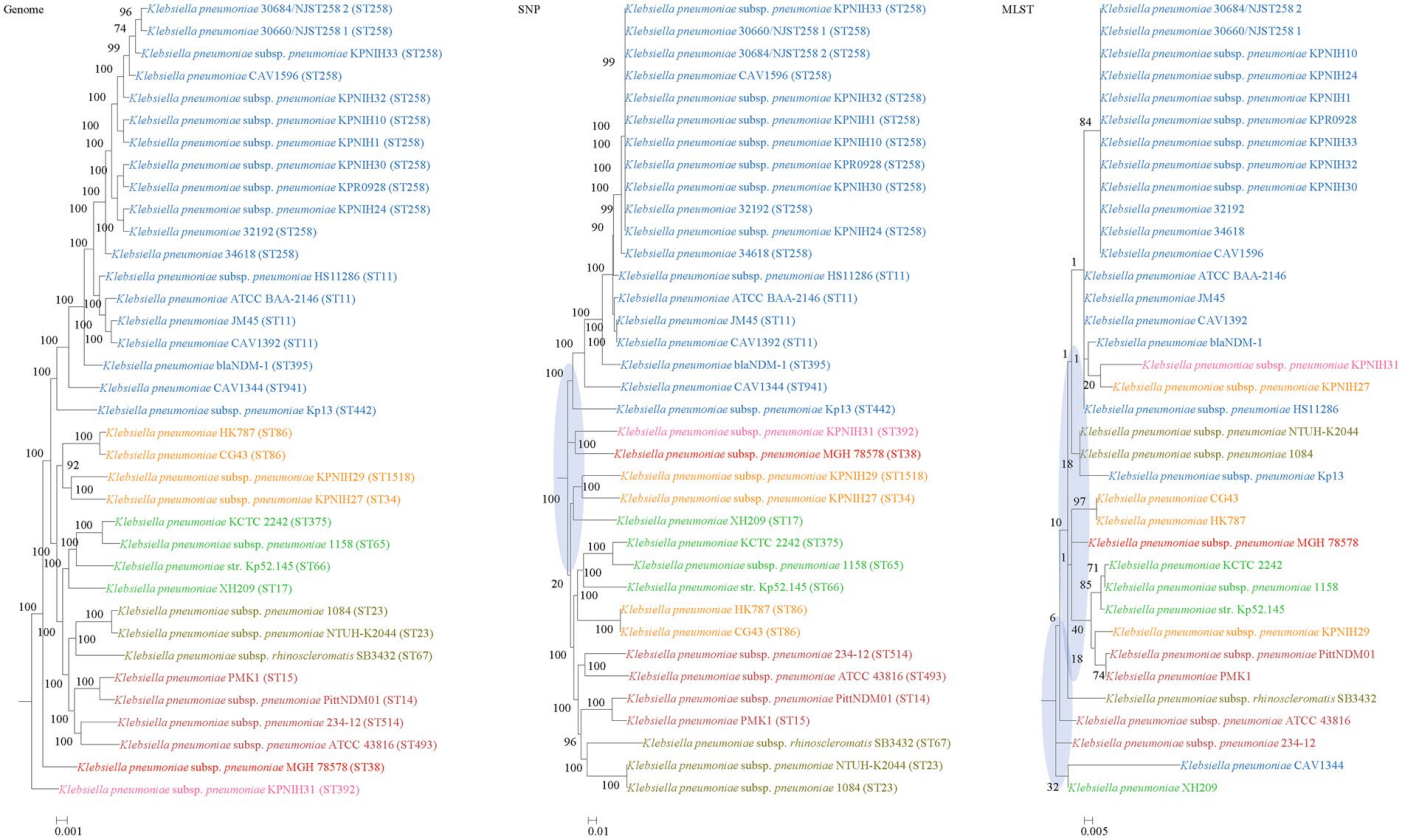
Comparison of phylogenetic trees constructed using genome data (left), SNP data (middle), and MLST data (right) for *Klebsiella pneumoniae*. Clusters that were manually selected based on the genome trees are illustrated in different colors. The unresolved polytomies are shaded in blue.

**Figure 7:**
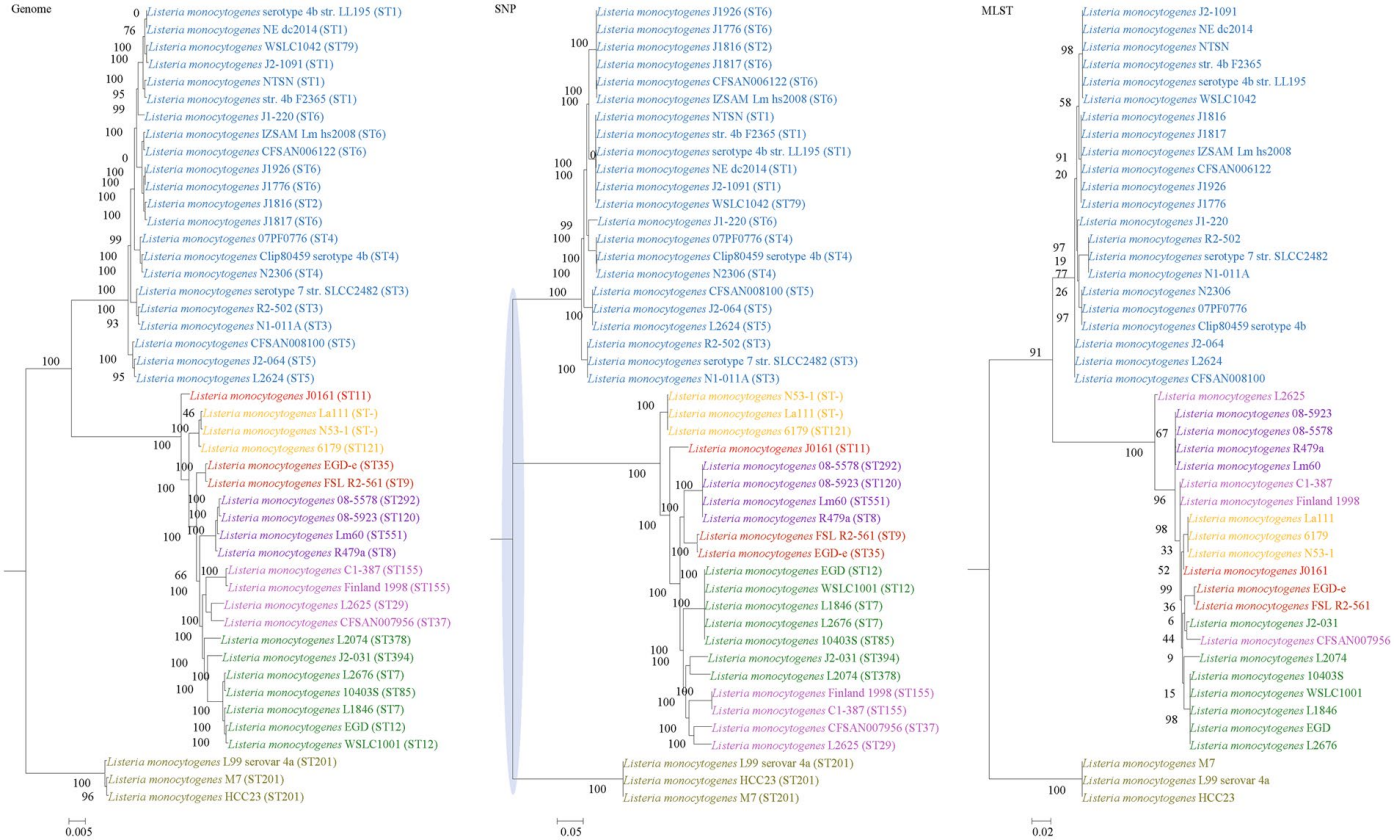
Comparison of phylogenetic trees constructed using genome data (left), SNP data (middle), and MLST data (right) for *Listeria monocytogenes*. Clusters that were manually selected based on the genome trees are illustrated in different colors. The unresolved polytomies are shaded in blue. A new sequence type is represented by a dash (“ST-”).

**Figure 8:**
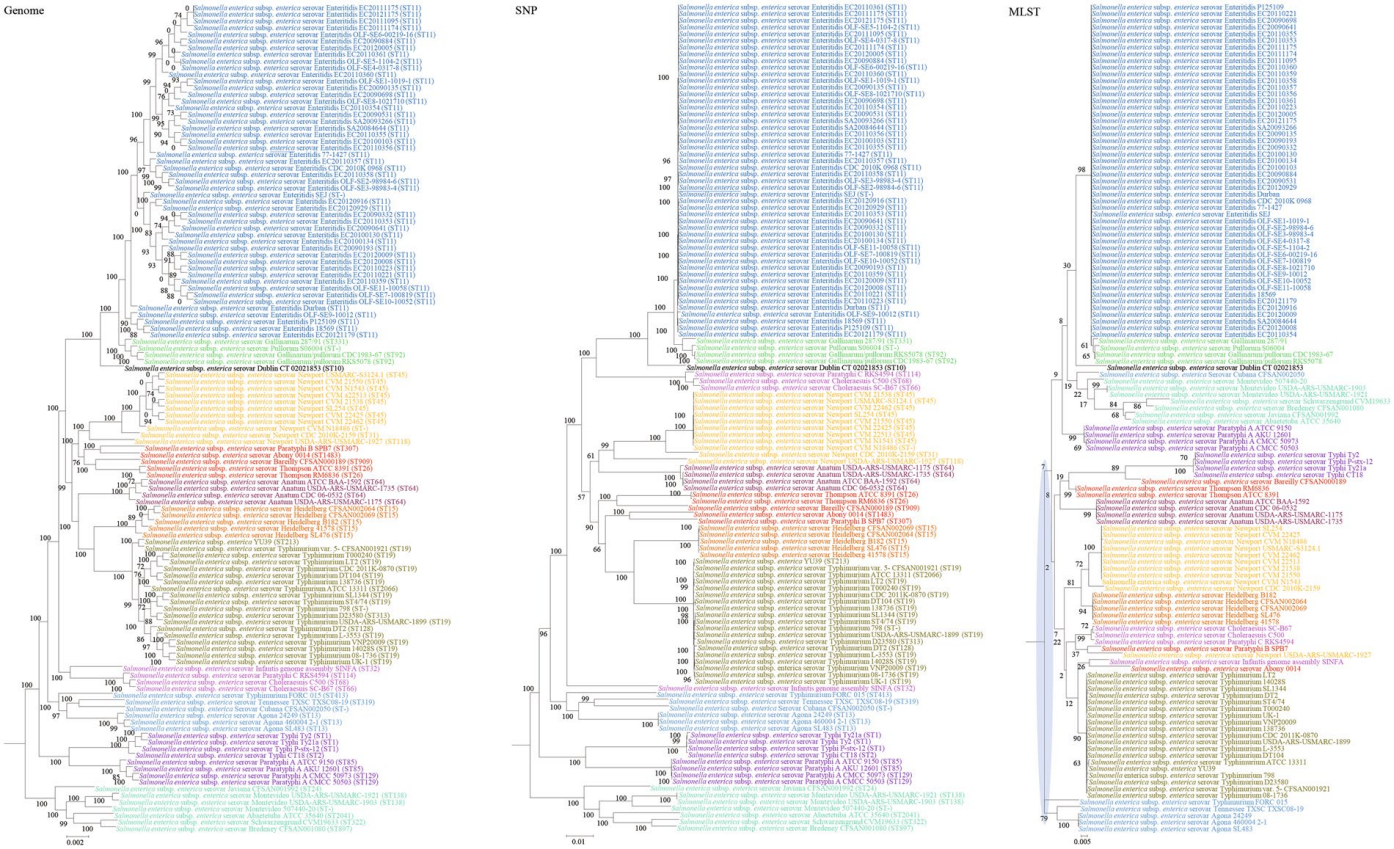
Comparison of phylogenetic trees constructed using genome data (left), SNP data (middle), and MLST data (right) for *Salmonella enterica*. Clusters that were manually selected based on the genome trees are illustrated in different colors. The unresolved polytomies are shaded in blue. A new sequence type is represented by a dash (“ST-”).

**Figure 9:**
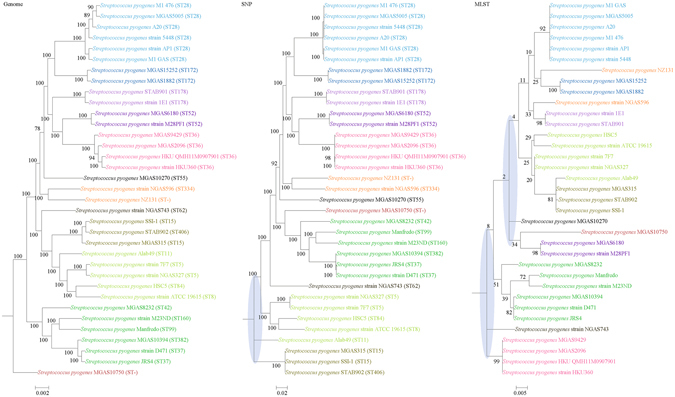
Comparison of phylogenetic trees constructed using genome data (left), SNP data (middle), and MLST data (right) for *Streptococcus pyogenes*. Clusters that were manually selected based on the genome trees are illustrated in different colors. The unresolved polytomies are shaded in blue. A new sequence type is represented by a dash (“ST-”).

**Figure 10:**
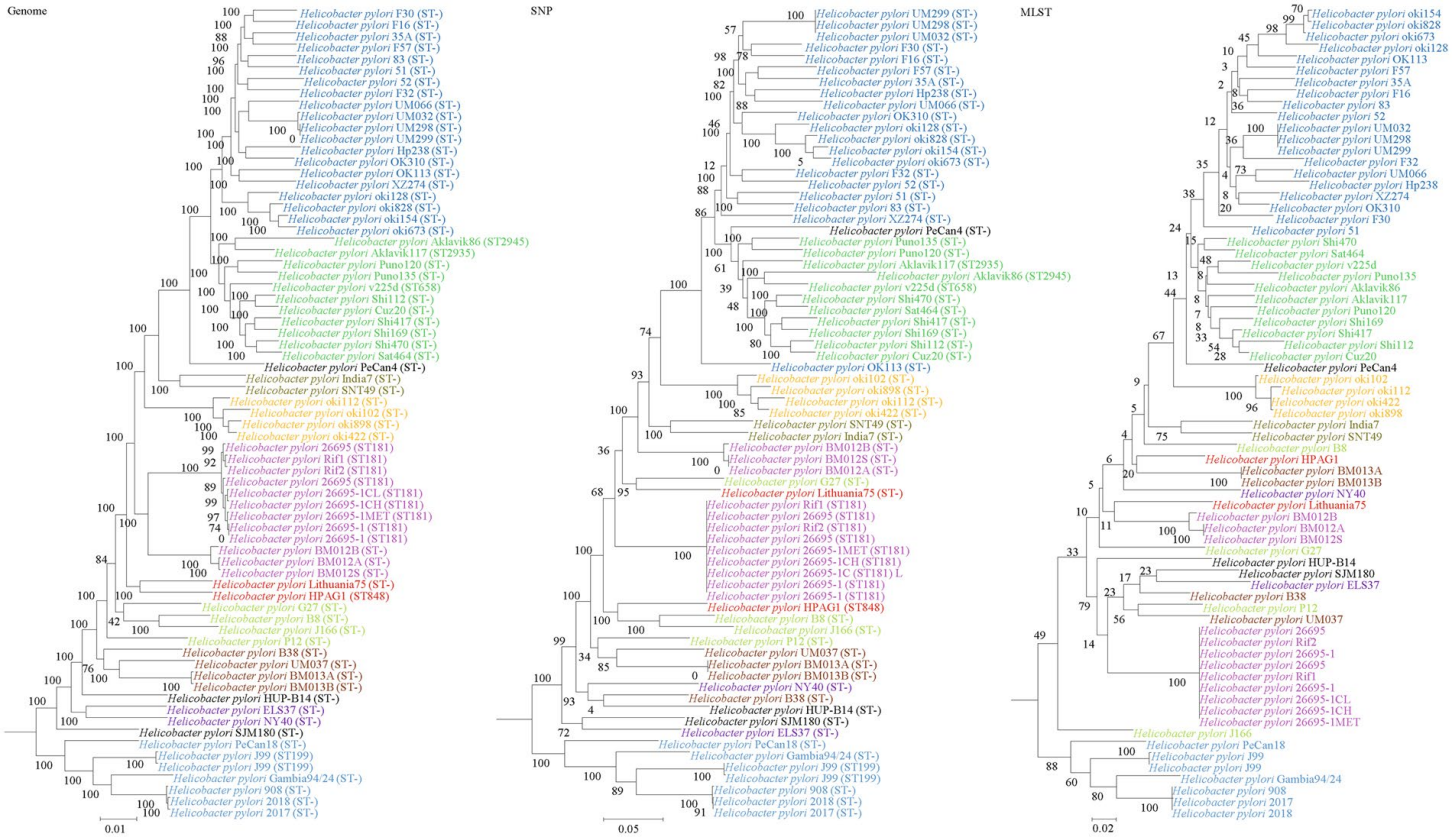
Comparison of phylogenetic trees constructed using genome data (left), SNP data (middle), and MLST data (right) for *Helicobacter pylori*. Clusters that were manually selected based on the genome trees are illustrated in different colors. A new sequence type is represented by a dash (“ST-”).

#### *Burkholderia pseudomallei*

In both the genome and SNP phylogenetic trees, NAU35A-3 was clustered with MSHR2243 and MSHR62 with significant branch support of 100; but in the MLST tree, NAU35A-3 was clustered with MSHR491. In both the genome and SNP phylogenetic trees, PB08298010 was clustered with 576 with significant branch support of 100; but in the MLST tree, PB08298010 was clustered with TSV 48. In both the genome and SNP phylogenetic trees, NCTC 13178 was clustered with MSHR1153 with significant branch support of 100; but in the MLST tree, they were phylogenetically distinct. In both the genome and SNP phylogenetic trees, 406e, BSR HBPUB10134a and PHLS 112 were clustered together with significant branch support of 100; but in the MLST tree, they were designated in separate clades.

#### *Klebsiella pneumonia*

In both the genome and SNP phylogenetic trees, SB3432 was grouped together with 1084 and NTUH K2044 into one cluster with significant branch support of 100; but in the MLST tree, SB3432 was phylogenetically distinct. In both the genome and SNP phylogenetic trees, 234–12 was clustered with ATCC 43816 with significant branch support of 100; but in the MLST tree, 234–12 was phylogenetically distinct.

#### *Salmonella enterica*

In both the genome and SNP trees, *S*. *enterica* serovar Heidelberg was clustered with *S*. *enterica* ser. Typhimurium, with significant branch support of 100; but in the MLST tree, *S*. *enterica* ser. Heidelberg was clustered with *S*. *enterica* ser. Newport. In both the genome and SNP trees, *S*. *enterica* ser. Cubana was clustered with *S*. *enterica* ser. Tennessee with significant branch support of 100 and 99 respectively; but in the MLST tree, *S*. *enterica* ser. Cubana was clustered with serovars including *S*. *enterica* ser. Montevideo, *S*. *enterica* ser. Schwarzengrund, *S*. *enterica* ser. Bredeney, *S*. *enterica* ser. Javiana and *S*. *enterica* ser. Abaetetuba. In both the genome and SNP trees, *S*. *enterica* ser. Abony was clustered with *S*. *enterica* ser. Paratyphi B with significant branch support of 100; but in the MLST tree, *S*. *enterica* ser. Abony was clustered with *S*. *enterica* ser. Infantis.

### Resolution and support for trees using different approaches

Both genome and SNP approaches yielded phylogenetic trees with strong support for most nodes. In contrast, for the trees inferred using MLST data, the bootstrap supports are generally low, with most of the nodes receiving less than 70% support (Figs 1–10). As for tree resolution, the genome phylogenetic trees are fully bifurcating; but both the SNP and MLST trees contain polytomies, with the MLST trees containing more polytomies than the SNP trees (Figs 1–9). For example, the phylogenetic trees are unresolved at the roots of the MLST trees of *B*. *pseudomallei*, *C*. *jejuni*, *S*. *pyogenes*, *K*. *pneumoniae*, *S*. *enterica*, *S*. *aureus* and *E*. *coli*.

### Statistical measurement of phylogenetic incongruence

For the Shimodaira-Hasegawa test, significant difference between the topologies of the genome tree and MLST tree were present for nine (*B*. *pseudomallei, C*. *jejuni, E*. *coli, H*. *pylori, K*. *pneumoniae, L*. *monocytogenes, S*. *aureus, S*. *enterica* and *S*. *pyogenes*) of the 10 bacterial species, and significant difference between the topologies of the SNP tree and MLST tree were present for all the 10 bacterial species (Table 2). Matching Clusters and R-F Clusters metrics were used to quantify the difference between the phylogenetic trees constructed using the three approaches. For both Matching Clusters and R-F Clusters metrics, the distances between the genome/SNP trees and MLST trees were larger than those between the SNP trees and genome trees (Table 3).

**Table 2:** Comparison by Shimodaira-Hasegawa test of log-likelihood scores between genome/SNP and MLST trees of the 10 bacterial species.

Species	MLST Tree –ln L	Genome vs MLST			SNP vs MLST		
		Genome Tree –ln L	Diff –ln L	P value	SNP –ln L	Diff –ln L	P value
*Burkholderia pseudomallei*	5994.26511	6164.70585	170.44074	0.002*	6190.62314	196.35803	0.002*
*Campylobacter jejuni*	7762.65452	8088.5789	325.40337	0.000*	8112.14513	349.49061	0.000*
*Chlamydia trachomatis*	7810.81726	7844.19200	33.37474	0.065	7857.47013	46.65286	0.032*
*Escherichia coli* 1	9412.75447	9845.31931	432.56484	0.000*	9836.13458	423.38011	0.000*
*Escherichia coli* 2	12615.10225	13363.74929	748.64704	0.000*	13428.07040	812.96814	0.000*
*Helicobacter pylori*	23840.02481	24459.54406	619.51925	0.000*	24423.92980	583.90499	0.000*
*Klebsiella pneumoniae*	5660.92269	5770.77356	109.85087	0.004*	5767.36723	106.44455	0.003*
*Listeria monocytogenes*	8418.29236	8563.19518	144.90282	0.002*	8541.07333	122.78098	0.006*
*Staphylococcus aureus*	8146.48997	8269.76377	123.27381	0.000*	8288.49580	142.00584	0.000*
*Salmonella enterica*	11447.21256	11769.73347	322.52092	0.000*	11787.43639	340.2283	0.000*
*Streptococcus pyogenes*	7481.09682	7682.21034	201.11352	0.000*	7622.24838	141.15156	0.001*

^*^P < 0.05.

**Table 3:** Tree distances among phylogenies inferred using different approaches.

Species	Genome vs MLST		SNP vs MLST		Genome vs SNP	
	Matching cluster	R-F cluster	Matching cluster	R-F cluster	Matching cluster	R-F cluster
*Burkholderia pseudomallei*	143	28	176	31	55	9
*Campylobacter jejuni*	156	25	176	24.5	62	7.5
*Chlamydia trachomatis*	436	57.5	466	58.5	102	18
*Escherichia coli* 1	564	76.5	491	72.5	307	27
*Escherichia coli* 2	531	74.5	564	70.5	307	27
*Helicobacter pylori*	257	45	212	42	171	32
*Klebsiella pneumoniae*	147	26	161	27	60	9
*Listeria monocytogenes*	112	28	124	28	48	8
*Staphylococcus aureus*	444	48	372	46.5	114	11.5
*Salmonella enterica*	1287	98.5	1242	97	214	31.5
*Streptococcus pyogenes*	95	17	127	17	72	7

## Discussion

In this study, we unambiguously showed that phylogeny inferred from MLST cannot fully represent genome phylogeny. Although MLST has been shown to be highly discriminatory and hence very useful for outbreak investigations and epidemiological surveillance of infections, it has always been controversial whether clustering and phylogeny inferred from the MLST gene loci can represent the real phylogeny of the strains. Despite this controversy, numerous publications on MLST, including those published in leading infectious disease and microbiology journals, did draw conclusions on clustering of the studied strains^10–14^. As complete genome sequencing has become less expensive with the next generation genome sequencing technologies such as the Roche 454 sequence and Illumina systems, the number of complete bacterial genomes sequenced has been rising exponentially in recent years. As a result, we are now able to construct genome phylogenetic trees of different strains of the same bacterial species. In the present study, we employed the phylogenetic tree constructed using concatenated blocks of homologous sequence shared between bacterial genomes as the gold standard of genome phylogeny to determine if the phylogenetic tree constructed using the MLST gene loci sequences, also extracted from the same set of complete genome sequences, can represent the phylogenetic relatedness of the bacterial strains. At the moment, more than 30 complete genomes are available for 10 highly important pathogenic bacteria, *B*. *pseudomallei, C*. *jejuni, C*. *trachomatis, E*. *coli, H*. *pylori, K*. *pneumoniae, L*. *monocytogenes, S*. *aureus, S*. *enterica* and *S*. *pyogenes*. Comparison of their genome trees and MLST trees by visual inspection revealed that their topologies are different. Major differences in strain clustering at more than one position were observed in the two trees for all 10 bacterial species (Figs 1–10).

In addition to the difference in topologies observed by visual inspection, the genome tree and MLST trees were shown to be incongruent according to three independent statistical tests for determining and quantifying the incongruence between the phylogenies, which included the Shimodaira-Hasegawa test, Matching Clusters and R-F Clusters metrics. The Shimodaira-Hasegawa test determines whether two tree topologies are equally well supported by the data, while the Matching Clusters metric calculates the smallest number of moves in order to transform one tree into the other and R-F Clusters metric calculates the number of different bipartitions between two trees. In this study, for the Shimodaira-Hasegawa test, incongruence between the genome tree and MLST tree were observed for all 10 bacteria except *C*. *trachomatis* (Table 2). For the Matching Clusters and R-F Clusters metrics, large distances were observed between the genome tree and MLST tree for all 10 bacteria (Table 3). All these indicate that phylogeny and clustering of bacterial strains using MLST trees may not represent their true phylogeny and clustering and therefore must be interpreted with great caution. For example, methicillin-resistant *S*. *aureus* with Panton-Valentine leucocidin ST772, associated with severe skin and soft tissue infections, was assigned to clonal complex 1^31–36^, as observed in the MLST tree in the present study (Fig. 1). However, the genome tree clearly showed that ST772 is more closely related to complex 5 than complex 1 with very high branch support of 100 (Fig. 1).

MLST fails to represent genome phylogeny because the seven genes used for MLST contain much less sequence information than the whole genome. In this study, the genome tree was constructed by whole genome alignment followed by extraction and concatenation of all locally collinear blocks. For example, for the genome tree for *S*. *aureus*, 618,392 bases per genome (21.8% of the genomes) were used for constructing the genome tree. On the other hand, only 3,186 bases, which belonged to seven independent genes in different parts of the genome, per genome (0.1% of the genomes) were used to construct the corresponding MLST tree. Hence, the MLST tree cannot fully represent genome phylogeny as it only contains 0.46% of the information used for genome tree construction. This is a problem in MLST, as one of these genes might even be subject of recombination, leading to conflicting results that do not correlate with the whole genome genetic information. In a typical MLST approach, MLST could be less vulnerable to recombination events by constructing trees from allelic profile data instead of total sequence similarity between strains. In recent years, the whole genome approach is vastly superior to using a single- or multiple-marker gene for examining phylogenetic relationships. The issues of recombination and horizontal gene transfer could be mitigated by using thousands or more genes in whole genome. It has also been observed that whole genome could provide much richer information than MLST and can be used to study microevolution in much finer detail in previous studies^37, 38^. In addition, the phylogenies inferred using MLST data are generally less resolved with low support, which is also likely due to the small number of informative sites. As genome sequencing has become much easier and cheaper than before, it should be performed for unambiguous typing of bacterial strains^16^. The whole genome sequencing approach provides the possibility to perform MLST on a genome-wide scale such as ribosomal MLST^39^, core genome MLST^40^ and whole genome MLST^41^ with increasing discriminatory power.

Interestingly, the SNP trees showed similar topologies to the genome trees by visual inspection and higher congruence to the genome trees compared to MLST trees. Genome-wide SNP trees were first used for analysis of *Bacillus anthracis* genomes^42, 43^ because of their coverage of the entire genome, relative simpler and less time consuming^44–46^. Although computational capacity in general doubles every 18 months, sequencing capability has been doubling every 6–9 months in recent years^47^. Therefore, it would be essential to look for less time consuming ways of analyzing genome phylogeny. For example, in the present study, constructing the whole genome tree for *S*. *aureus* took about 31 hours with a Xeon 5690 CPU and 48 GB memory, whereas only 3 minutes was used for constructing the SNP tree. As the conclusions drawn from SNP trees are consistent with the genome trees, the SNP trees may be considered as an alternative in whole genome epidemiology studies, particularly in situations where computational resources and time are limited.

## Electronic supplementary material

Supplementary Table S1
