# Supplementary material for: Failure of phylogeny inferred from multilocus sequence typing to represent bacterial phylogeny
Source: Sci Rep. 2017 Jul 3;7:4536. doi: 10.1038/s41598-017-04707-4 (PMC5495804; doi:10.1038/s41598-017-04707-4)
Supplement: Supplementary file 1 — Supplementary Table S1 [file 41598_2017_4707_MOESM1_ESM.pdf]

Failure of phylogeny inferred from multilocus sequence typing to represent bacterial phylogeny

Alan K. L. Tsang[1], Hwei Huih Lee[1], Siu-Ming Yiu[2], Susanna K. P. Lau[1,3,4,5,6]*, Patrick C. Y. Woo[1,3,4,5,6]*

Supplementary Table S1. Bacteria used in study

Supplementary Table S1

Bacteria and strains	GenBank accession no.	Remarks
Burkholderia pseudomallei		
K96243	BX571965	Isolated from a 34-year-old female diabetic patient with melioidosis in Thailand in 1996
1710b	CP000124	Opportunistic human pathogen, isolated from blood culture of a 55-year-old diabetic patient with primary melioidosis in Thailand in 1999
668	CP000570	Isolated from the blood of a 53 year old male patient with severe melioidosis encephalomyelitis in Darwin, Australia in 1995.
1106a	CP000572	Isolated in 1993 in Ubon Ratchathani, Thailand from pus aspirated from liver abscess from a 23-year old female rice farmer
1026b	CP002833	Isolated from the blood of a 29-year-old female patient with septicemic melioidosis with skin, soft tissue, spleen and joint involvement
BPC006	CP003781	Isolated from blood of patient with type I diabetes and multiple abscesses, a melioidosis patient in Hainan, China
MSHR305	CP006470	Isolated in 1994 during an autopsy of a fatal melioidosis encephalomyelitis case at the Royal Darwin Hospital, Northern Territory, Australia
NCTC 13179	CP003976	Isolated from human skin ulcer in Australia
NCTC 13178	CP004001	Isolated from human post mortem brain in Australia
NAU20B-16	CP004003	Isolated from soil in Australia
MSHR511	CP004023	Isolated from throat of goat in Australia
MSHR146	CP004042	Isolated from right udder of goat in Australia
MSHR520	CP004368	Isolated from human blood in Australia
BDP	CP009209	Isolated from human brain in Darwin, Northern Territory, Australia
BSR	CP009128	-
HBPUB10303a	CP008894	Isolated from sputum of a 40 year old male with melioidosis through tracheal suction in Thailand
HBPUB10134a	CP008911	Isolated from human sputum through tracheal suction of human in Thailand
MSHR5858	CP008892	Isolated from human sputum in Australia
MSHR5848	CP008909	Isolated from human sputum in Australia
MSHR5855	CP008784	Isolated from human sputum in Australia
BGR	CP008834	Collected from Thailand

Mahidol-1106a	CP008781	Collected from Thailand
MSHR1655	CP008780	Isolated from a patient with melioidosis in Darwin, Australia in 2000
576	CP008777	isolated from a 49 year old woman suffering from fatal melioidosis in Thailand
PHLS 112	CP009585	A human isolate obtained from northeast Thailand in 1992
Pasteur 52237	CP009899	Isolated from Vietnam
NAU35A-3	CP004377	Isolated from soil in Australia
MHSR62	CP009235	Human clinical isolate from Australia
B03	CP009151	Isolated from environmental sample in Australia
TSV 48	CP009161	Isolated from environmental sample in Australia
K 42	CP009162	Isolated from environmental sample in Australia
A79A	CP009165	Isolated from environmental sample in Australia
vgh07	CP010973	Isolated from a melioidosis patient with arthro-osteomyelitis in Taiwan.
406e	CP009298	Collected from human sample in Thailand
MSHR840	CP009474	Isolated from human brain tissue in Ipswitch, Australia
MSHR491	CP009485	Isolated from community water supply storage tank at Northern Territory, Australia
MSHR2543	CP009478	Isolated from Australia
7894	CP009535	A human isolate obtained from Ecuador in 1962
MSHR668	CP009545	Isolated from the blood of a 53 year old male patient with severe melioidosis encephalomyelitis in Darwin, Australia in 1995
PB08298010	CP009551	Isolated from human clinical sample from Arizona, USA
MSHR346	CP008764	Isolated from sputum of a melioidosis patient admitted to Royal Darwin Hospital, Northern Territory, Australia in 1995
MSHR2243	CP009270	Isolated from clinical human sample in Australia
MSHR1153	CP009271	Isolated from clinical human sample in Australia
Campylobacter jejuni		
NCTC 11168	AL111168	Subspecies jejuni, isolated from clinical sample in the UK in 1977
RM1221	CP000025	Isolated from chicken carcasses at the retail level in the US
81-176	CP000538	Subspecies jejuni, isolated from campylobacteriosis specimens related to consumption of raw milk at a Minnesota farm in 1985
81116	CP000814	Subspecies jejuni, isolated from a waterborne outbreak in 1982 and used as a laboratory strain
IA3902	CP001876	Subspecies jejuni, isolated from an aborted ovine fetus
M1	CP001900	Subspecies jejuni, isolated from the diarrheic stools of one research team member 9 days after visiting a poultry processing plant
CG8421	CP005388	Subspecies jejuni, clinical isolate from a patient in Thailand

S3	CP001960	Subspecies jejuni, poultry isolate, originally cultured from the feces of a chicken
PT14	CP003871	Subspecies jejuni, used to isolate and propagate bacteriophages from environmental samples
NCTC 11168-BN148	HE978252	Subspecies jejuni, isolated in 1979 from the collection of the Centers for Disease Control and Prevention
32488	CP006006	Isolated from human sample in US
00-2538	CP006707	Subspecies jejuni, isolated from stool sample in Canada
00-2544	CP006709	Subspecies jejuni, isolated from stool sample in Canada
00-2426	CP006708	Subspecies jejuni, isolated from stool sample in Canada
00-2425	CP006729	Subspecies jejuni, isolated from stool sample in Canada
4031	HG428754	Isolate from contaminated tap water
R14	CP005081	Subspecies jejuni
MTVDSCj20	CP008787	Subspecies jejuni, isolated from the cecal contents of a farm-raised chicken
F38011	CP006851	Subspecies jejuni, isolated from human sample in Arizona
YH001	CP010058	Subspecies jejuni, isolated from beef liver in Philadelphia, US
NCTC 11168-K12E5	CP006685	Subspecies jejuni
NCTC 11168-Kf1	CP006686	Subspecies jejuni
NCTC 11168-mcK12E5	CP006687	Subspecies jejuni
NCTC 11168-mfK12E5	CP006688	Subspecies jejuni
NCTC 11168-GSv	CP006689	Subspecies jejuni
00-1597	CP010306	Subspecies jejuni, isolated from a 34 year old patient with gastroenteritis stool sample in Alberta, Canada
00-6200	CP010307	Subspecies jejuni, isolated from a 29 year old patient with gastroenteritis stool sample in Ontario, Canada
01-1512	CP010072	Subspecies jejuni, isolated from a 1 year old infant New Brunswick, stool sample in Canada
00-0949	CP010301	Subspecies jejuni, isolated from a 26 year old patient stool sample in Quebec, Canada
35925B2	CP010906	Subspecies jejuni, clinical isolate from Chicago, US
269.97	CP000768	Subspecies doylei, isolated from a blood sample of human bacteremia in South Africa

Chlamydia trachomatis		
D/UW-3/CX	AE001273	Serovar D, isolated from the cervix of an asymptomatic female.
L2/434/Bu	AM884176	Serovar L2, isolated from an inguinal bubo of a Lymphogranuloma venereum case in California
A/HAR-13	CP000051	Serovar A, oculotropic trachoma isolate
B/TZ1A828/OT	FM872307	Serovar B, ocular isolate isolated in Tanzania
B/Jali20/OT	FM872308	Serovar B, ocular isolate isolated in the Gambia
L2b/UCH-1/proctitis	AM884177	Serovar L2b, isolated from a rectal swab of a 49-year-old men with proctitis who have sex with men, and was HIV positive and Hepatitis C negative in London in 2006
D-EC	CP002052	Serovar D, a laboratory reference strain
E/150	CP001886	Serovar E, a clinical isolate from rectum
G/9768	CP001887	Serovar G, a clinical isolate from rectum
G/11222	CP001888	Serovar G, a clinical isolate from cervix
G/11074	CP001889	Serovar G, a clinical isolate from rectum
E/11023	CP001890	Serovar E, a clinical isolate from cervix
G/9301	CP001930	Serovar G, a clinical isolate from urethra
D-LC	CP002054	Serovar D, a clinical isolate
Sweden2	FN652779	Serovar E, isolated in McCoy cells in October 2006 from a urethral sample from a Swedish man, suffering from symptomatic chlamydial urethritis
Lc2	CP002024	Serovar L2, isolated from a men with severe hemorrhagic proctitis who have sex with men
A2497	CP002401	Serovar A, isolated from a 2.5-year-old with intense active trachoma; Rombo District, Kilimanjaro Region, Tanzania; 2001
A2497 serovarA	FM872306	Serovar A, a plasmid-deficient strain made from a 2.5-year-old with intense active trachoma in Tanzania
E/SW3	HE601801	Serovar E, urogenital isolates from Malmö, Southern Sweden, in 2002
F/SW4	HE601804	Serovar F, urogenital isolates from Malmö, Southern Sweden, in 2002
F/SW5	HE601805	Serovar F, urogenital isolates from Malmö, Southern Sweden, in 2002
A/363	HE601796	Serovar A, ocular isolate from Kahe Mpya, Rombo district, Tanzania
A/7249	HE601797	Serovar A, ocular isolate from Kahe Mpya, Rombo district, Tanzania
E/Bour	HE601870	Serovar E, ocular isolate from California, USA
Ia/SotonIa1	HE601808	Serovar Ia, endocervix isolate from Southampton, UK
Ia/SotonIa3	HE601809	Serovar Ia, endocervix isolate from Southampton, UK
L1/440/LN	HE601950	Serovar L1, lymph node isolate from California, USA
L1/115	HE601952	Serovar L1, LGV patient isolate from Ciskei, South Africa

L1/224	HE601953	Serovar L1, LGV patient isolate from Transkei, South Africa
L2/25667R	HE601954	Serovar L2, Rectal biopsy isolate from USA
L2b/8200/07	HE601795	Serovar L2b, rectum isolate from Sweden
L2b/UCH-2	HE601956	Serovar L2b, rectum isolate from London, UK
L2b/795	HE601949	Serovar L2b, rectum isolate from Bordeaux, France
L3/404/LN	HE601955	Serovar L3, lymph node isolate from California, USA
L2/434/Bu(i)	CP003963	Serovar L2, isolated from an inguinal bubo of a Lymphogranuloma venereum case in California
L2/434/Bu(f)	CP003965	Serovar L2, isolated from an inguinal bubo of a Lymphogranuloma venereum case in California
C/TW-3	CP006945	Serovar C, isolated in Taiwan in 1959 from the human conjunctiva
F/1-93	CP006671	Serovar F, isolated in Washington, USA from human endo-cervical swab
F/6-94	CP006673	Serovar F, isolated in Washington, USA from human endo-cervical swab
F/11-96	CP006674	Serovar F, isolated in Washington, USA from human endo-cervical swab
D/13-96	CP006676	Serovar D, isolated in Washington, USA from human endo-cervical swab
J/31-98	CP006680	Serovar J, isolated in Washington, USA from human endo-cervical swab
L2b/CS19/08	CP009923	Serovar L2b, isolated in Lisbon, Portugal from human LGV proctitis
L2b/CS784/08	CP009925	Serovar L2b, isolated in Lisbon, Portugal from human LGV proctitis
A/5291	HE601810	Serovar A, ocular isolate from Tanzania
D/SotonD1	HE601798	Serovar D, endocervix isolate from Southampton, UK
D/SotonD5	HE601799	Serovar D, endocervix isolate from Southampton, UK
D/SotonD6	HE601800	Serovar D, endocervix isolate from Southampton, UK
E/SotonE4	HE601802	Serovar E, endocervix isolate from Southampton, UK
E/SotonE8	HE601803	Serovar E, endocervix isolate from Southampton, UK
F/SotonF3	HE601806	Serovar F, endocervix isolate from Southampton, UK
G/SotonG1	HE601807	Serovar G, endocervix isolate from Southampton, UK
K/SotonK1	HE601794	Serovar K, endocervix isolate from Southampton, UK
L2b/LST	HE601958	Serovar L2b, rectum isolate from Tourcoing, France
L2b/CV204	HE601960	Serovar L2b, rectum isolate from Paris, France
L2b/Ams1	HE601959	Serovar L2b, penile ulcer isolate from Ne
L2b/Ams2	HE601961	Serovar L2b, anus isolate from Netherlands
L2b/Ams3	HE601962	Serovar L2b, anus isolate from Netherlands
L2b/Ams4	HE601964	Serovar L2b, anus isolate from Netherlands

L2b/Ams5	HE601965	Serovar L2b, anus isolate from Netherlands
L2b/Canada1	HE601963	Serovar L2b, rectum isolate from Vancouver, Canada
L2b/Canada2	HE601957	Serovar L2b, rectum isolate from Vancouver, Canada
IU888	HF562300	Serovar I, isolate from a 19-year-old woman 10 months after treatment with tetracycline and erythromycin for acute salpingitis
IU824	HF562298	Serovar I, isolate from the endometrium of a 27- year-old woman 5 months after tetracycline and 11 months after cephalosporin treatment
F/2-93	CP006672	Serovar F, endo-cervical swab from Washington, USA
E/12-94	CP006675	Serovar E, endo-cervical swab from Washington, USA
D/14-96	CP006677	Serovar D, endo-cervical swab from Washington, USA
Ia20-97	CP006678	Serovar Ia, endo-cervical swab from Washington, USA
J27-97	CP006679	Serovar J, endo-cervical swab from Washington, USA
E/C599	HE605379	Serovar E, isolate from a 26-year-old asymptomatic male
F/SWFPminus	HE605380	Serovar F, cervical isolate from Malmo, Sweden, in 1995
Escherichia coli		
042	FN554766	EAEC, used for comparative analysis
536	CP000247	UPEC, O6:K15:H31, isolated from a patient with acute pyelonephritis
55989	CU928145	EAEC, clinical enteroaggregative isolate
ABU 83972	CP001671	causes an asymptomatic bacteriuria
APEC O1	CP000468	APEC, O1:K1:H7, avian pathogenic strain and causes respiratory, blood, and enteric infections primarily in poultry
ATCC 8739	CP000946	K-12 derivative, lacking the major porin OmpC
REL606	CP000819	Commensal, strain B, used in long term evolution experiments to study rates of mutation and adaption
BL21(DE3)	CP001509	Commensal, strain B, a derivative of E. coli strain B
BL21(DE3)	AM946981	Commensal, strain B, derived from strain B
BL21-Gold(DE3)pLysS AG	CP001665	Commensal, strain B, a derivative of E. coli strain B
BW2952	CP001396	K-12 derivative, used for comparative analysis
CFT073	AE014075	UPEC, O6:K2:H1
DH1	CP001637	K12 derivative, a common laboratory strain
E24377A	CP000800	ETEC, O139:H28
HS	CP000802	Commensal, O9, a human commensal
IAI1	CU928160	Commensal, O8, a serogroup O8 strain
IAI39	CU928164	UPEC, O7:K1, a Serovar O7:K1 strain from a urinary tract infection
IHE3034	CP001969	ExPEC, O18:K1:H7, a neonatal meningitis-associated strain isolated in Finland in 1976

12009	AP010958	EHEC, O103:H2
11128	AP010960	EHEC, O111:H-
E2348/69	FM180568	EPEC, O127:H6
EC4115	CP001164	EHEC, O157:H7, used for comparative analysis
Sakai	BA000007	EHEC, O157:H7, isolated in a 1997 outbreak in Sakai, Japan
TW14359	CP001368	EHEC, O157:H7, isolated from spinach during the E. coli outbreak in 2006
11368	AP010953	EHEC, O26:H11, a human enterohemorrhagic E. coli which attaches to and effaces cells in the large intestine
CB9615	CP001846	O55:H7, isolated from an infant with diarrhea in Germany in 2003
S88	CU928161	ExPEC, O45:K1:H7, a Serovar O45:K1 strain isolated from a case of neonatal meningitis
SE11	AP009240	Commensal, O152:H28, for comparative genome analysis
SE15	AP009378	Commensal, O150:H5, for comparative genome analysis
SMS-3-5	CP000970	Environmental isolate, isolated from a toxic-metal contaminated site, Shipyard Creek, Charleston, South Carolina, highly resistant to a number of antibiotics
UM146	CP002167	-
UMN026	CU928163	UPEC, O7:K1, a Serovar O7:K1 clinical isolate
UTI89	CP000243	UPEC, isolated from a woman with uncomplicated cystitis
K-12 substrain W3110	AP009048	Commensal, K-12, closely related to MG1655 and is descended from K-12
NRG 857C	CP001855	AIEC, O83:H1, clinical isolate from the ileum of a Crohn's Disease patient
2011C-3493	CP003289	EAEC, O104:H4, isolated from US citizen afflicted with hemolytic uremic syndrome after travel to Germany during the 2011 *E. coli* outbreak
KO11	CP002516	Constructed for production of ethanol from hexose and pentose sugars in hemicellulose hydrolysates by inserting the *Zymomonas mobilis* genes encoding *pdc* and *adhB* during fermentation
W	CP002185	Isolated from soil of a cemetery near Rutgers University in 1943, show sensitivity to broad range of antibiotics especially streptomycin
ETEC H10407	FN649414	ETEC, prototypical strain of *E. coli*, which reproducibly elicits diarrhea in human volunteer studies. Isolated from an adult with cholera-like symptoms in the course of an epidemiologic study in Dacca, Bangladesh, prior to 1973.
UMNK88	CP002729	ETEC, isolated in 2007 from a farm in Minnesota
CE10	CP003034	NMEC, O7:K1, isolated from the CSF of a neonate with meningitis,

		representative of group 2 isolates from CSF, carry a complete gene set encoding the type III secretion system, which involved in the invasion and intracellular survival of the bacterium in human brain microvascular endothelial cells
'clone D i2'	CP002211	*E.coli* that causes urinary tract infection isolated from a women in 2005
'clone D i14'	CP002212	*E.coli* that causes urinary tract infection isolated from a dog
RM12579	CP003109	EPEC, multidrug-resistant, isolated in December 1974 from the urine of a child less than 5 years old by the California Department of Public Health
P12b	CP002291	O15:H17, non-pathogenic *E. coli* reference strains
KO11FL	CP002970	New version of *E.coli* KO11 (ATCC 55124) engineered to produce ethanol by chromosomal insertion of the *Zymomonas mobilis pdc* and *adhB* genes into *E. coli* W (ATCC 9637)
W	CP002967	Isolated from soil of a cemetery near Rutgers University in 1943, show sensitivity to broad range of antibiotics especially streptomycin
Xuzhou21	CP001925	EHEC, isolated from an hemolytic uremic syndrome patient from the 1999 Xuzhou outbreak
DH1 ME8569	AP012030	A commonly used laboratory strain as model organism strain derived from strain DH1 from Japan
LF82	CU651637	AIEC, isolated from a patient with Crohn's disease
ST131 strain EC958	HG941718	O25b:H4, a leading multi-drug resistant pathogen causing urinary tract and bloodstream infections in hospitals and the community
2009EL-2050	CP003297	EAEC, O104:H4, isolated from a human patient during 2009 bloody diarrhea outbreak in the Republic of Georgia
2009EL-2071	CP003301	EAEC, O104:H4, isolated from a human patient during 2009 bloody diarrhea outbreak in the Republic of Georgia
APEC O78	CP004009	APEC, isolated from the lung of a turkey clinically diagnosed with colibacillosis
K-12 substrain MDS42	AP012306	Non-pathogenic laboratory strain
LY180	CP006584	Laboratory strain engineered to ferment sugars (glucose and xylose) into ethanol, derived from the widely used KO11 strain
C321.deltaA	CP006698	First Genomically Recoded Organism
JJ1886	CP006784	UPEC, multidrug-resistant. Associated with recurrent urinary tract infections, pyelonephritis, and sepsis. Obtained in 2007 in the United States from a patient with fatal urosepsis.
K-12 substrain MC4100	HG738867	Laboratory strain derived from *E. coli* K12 strains MO and Hfr 3000 U169

RM13514	CP006027	STEC, O145:H28, clinical isolate and linked to the 2010 multi-state outbreak of *E. coli* O145 infection associated with consumption of shredded romaine lettuce in US
RM13516	CP006262	STEC, O145:H28, isolated from a patient in Belgium during a 2007 outbreak of infection associated with consumption of ice cream produced in a farm
ST540	CP007265	nitrofurantoin-sensitive *E. coli* from stool samples of Belgian outpatients with urinary tract infection
ST540	CP007390	nitrofurantoin-sensitive *E. coli* from stool samples of Belgian outpatients with urinary tract infection
ST540	CP007391	nitrofurantoin-sensitive *E. coli* from stool samples of Belgian outpatients with urinary tract infection
ST2747	CP007392	nitrofurantoin-sensitive *E. coli* from stool samples of Belgian outpatients with urinary tract infection
ST2747	CP007393	nitrofurantoin-sensitive *E. coli* from stool samples of Belgian outpatients with urinary tract infection
ST2747	CP007394	nitrofurantoin-sensitive *E. coli* from stool samples of Belgian outpatients with urinary tract infection
RM12761	CP007133	O145:H28. Non-O157 STEC *E. coli* isolated from ice cream during a 2007 ice cream-associated outbreak in Belgium
RM12581	CP007136	STEC, O145:H28. Non-O157 *E. coli* isolated from bagged romaine lettuce during a 2010 U.S. lettuce-associated outbreak
Nissle 1917	CP007799	Probiotic *E. coli* isolated in 1917 based on its potential to protect from presumably infectious gastroenteritis
KLY	CP008801	-
SS17	CP008805	EHEC, O157:H7. Obtained from the recto-anal junction of a cow, a super-shedder isolate
EDL933	CP008957	EHEC, O157:H7. Isolated from Michigan ground beef linked to the outbreak in 1982 involving contaminated hamburgers
ATCC 25922	CP009072	Isolated from a clinical sample in Seattle, Washington in 1946, and is often used in quality control testing
BW25113	CP009273	Common laboratory strain created in the laboratory of Barry L. Wanner
ER2796	CP009644	Derived from JC1552, a DNA Methyltransferase-Deficient Laboratory K-12 derivative
K-12 strain ER3413	CP009789	Sequenced by the Blattner laboratory because it approximates wild-type *E. coli* and has been maintained as a laboratory strain with minimal genetic manipulation
RM9387	CP009104	STEC, O104:H7, isolated from cattle feces

94-3024	CP009106	STEC, O104:H21, caused an outbreak of hemorrhagic colitis in Montana in 1994 linked to contaminated milk
K-12 substrain MG1655	CP009685	Commensal, K-12, a non-pathogenic strain MG1655 approximates wild-type *E. coli*
SS52	CP010304	STEC, O157:H7, obtained from the recto-anal junction of a cow at Midwestern United States, a super-shedder isolate
APEC IMT5155	CP005930	APEC, O2:K1:H5, isolated from the internal organs of a laying hen with clinical symptoms of septicemia.
6409	CP010371	-
789	CP010315	APEC, O78, isolated from the bone marrow of a chicken with acute colisepticaemia
Santai	CP007592	EHEC, O157:H16, special pathogenic isolation in clinical healthy adult laying duck
1303	CP009166	O70:H32, isolated from udder secretions of a cow with clinical mastitis
C41(DE3)	CP010585	“Walker strains” used in membrane protein overexpression
ECC-1470	CP010344	Ont:Hnt. isolated from a persistent infection cow
BL21 (TaKaRa)	CP010816	Common laboratory strains for recombinant protein production
MNCRE44	CP010876	ExPEC, ST131, Isolated in 2012 from the sputum sample from a patient with multiple comorbidities in Minnesota
HUSEC2011	HF572917	EHEC, Isolated from patients with hemolytic uremic syndrome
VR50	CP011134	Asymptomatic bacteria isolate associated with urinary tract infection
CI5	CP011018	UPEC, clinical pyelonephritis isolate, used as *in vitro* cell culture models and *in vivo* murine infection models
C227-11	CP011331	EAEC, O104:H4. Isolated on 24 May 2011 from a German patient at Hvidovre University Hospital in Denmark during the O104:H4 outbreak
SEC470	CP007594	ETEC, O4, isolated from a 39-day-old piglet with diarrhea at Jiangxi Province, China
SQ37	CP011320	A ribosomal deletion construct of *E. coli*
SQ88	CP011321	A chromosome of the five rRNA operon knockout E. coli strain
SQ110	CP011322	A ribosomal deletion construct of *E. coli* MG1655
SQ171	CP011323	A ribosomal deletion construct of *E. coli*
SQ2203	CP011324	A ribosomal deletion construct of *E. coli*
CFSAN029787	CP011416	Isolate of human stool sample from Milan
PMV-1	HG428755	ExPEC, used as a model for peritonitis in mice and ExPEC infections
GM4792 Lac+	CP011342	Common laboratory *E. coli* strain
PCN033	CP006632	ExPEC, isolated from brain of swine with meningitis
PCN061	CP006636	ExPEC, isolated from extraintestinal site of diseased pig
ACN001	CP007442	APEC, causes zoonotic diseases.

NCM3722	CP011495	A prototrophic K-12 strain with robust physiologic phenotypes
C43(DE3)	CP011938	“Walker strains” used in membrane protein overexpression
DH1Ec095	CP012125	K12 derivative, a common laboratory strain
DH1Ec104	CP012126	K12 derivative, a common laboratory strain
DH1Ec169	CP012127	K12 derivative, a common laboratory strain
Helicobacter pylori		
26695	AE000511	Isolated from a patient in the United Kingdom who had gastritis before 1987
J99	AE001439	Isolated in 1994 in the USA from a patient with duodenal ulcer
51	CP000012	Isolated from a duodenal ulcer patient who had undergone a gastrointestinal examination in a hospital in South Korea
HPAG1	CP000241	Isolated from a Swedish patient with chronic atrophic gastritis, the precursor to gastric adenocarcinoma
Shi470	CP001072	Clinical isolate from gastric antrum from Amerindian resident of remote Amazonian village of Shimaa, Peru
G27	CP001173	Isolated from an endoscopy patient in Italy
P12	CP001217	Isolated from a duodenal ulcer patient in Germany in 1992
52	CP001680	Strain from Korea used for comparative study
B38	FM991728	Isolated from a 62-year-old man suffering from MALT lymphoma in France
v225d	CP001582	Isolated from a gastric antral biopsy specimen from a Piaroa Amerindian who underwent a gastroscopy and was found to have acute superficial gastritis
908	CP002184	Isolated from an African patient living in France, who suffered from duodenal ulcer disease
SJM180	CP002073	Isolated from an Amerindian resident of San Juan Miraflores shantytown in Lima Peru with gastritis
PeCan4	CP002074	Isolated from a Peruvian gastric cancer patient
Cuz20	CP002076	isolated from an Amerindian resident of the remote Amerindian village from Monte Carmelo near Cuzco, Peru
Sat464	CP002071	Isolated from Amerindian resident from Satipo region, Peru
35A	CP002096	Isolated from human stomach, reference genome for Human Microbiome Project
India 7	CP002331	Clinical isolate
Gambia94/24	CP002332	Clinical isolate
Lithuania75	CP002334	Clinical isolate
2017	CP002571	Isolated on 2003 from antrum of an African patient who suffered from

		recrudescent duodenal ulcer disease in France
2018	CP002572	Isolated on 2003 from corpus of an African patient who suffered from recrudescent duodenal ulcer disease in France
B8	FN598874	Isolated from a human gastric ulcer patient, was adapted to Mongolian gerbil
83	CP002605	Isolated from human stomach, reference genome for Human Microbiome Project
Puno120	CP002980	Clinical isolate from gastric antrum from Amerindian resident of Puno region in Peru
Puno135	CP002982	Clinical isolate from gastric antrum from Amerindian resident of Puno region in Peru
SNT49	CP002983	Isolate from Santal tribe in India
ELS37	CP002953	Isolate from gastric cancer patient from El Salvador rural area
HUP-B14	CP003486	Clinical isolate from resident of Spain
XZ274	CP003419	Isolated from the stomach of a female Tibetan patient with gastric cancer in China
F16	AP011940	Isolated from patients with gastritis in Fukui, Japan
F30	AP011941	Isolated from patients with duodenal ulcer in Fukui, Japan
F32	AP011943	Isolated from patients with intestinal type gastric cancer in Fukui, Japan
F57	AP011945	Isolated from patients with diffuse type gastric cancer in Fukui, Japan
Shi417	CP003472	Isolated from gastric antrum of Amerindian volunteer from remote Shimaa village in Amazon region in Peru
Shi169	CP003473	Isolated from gastric antrum of Amerindian volunteer from remote Shimaa village in Amazon region in Peru
Shi112	CP003474	Isolated from gastric antrum of Amerindian volunteer from remote Shimaa village in Amazon region in Peru
PeCan18	CP003475	Clinical isolate from gastric antrum from resident of Lima, Peru
26695	CP003904	Isolated from a patient in the United Kingdom with gastritis
Rif1	CP003905	Rifampin-resistant strain selected from H. pylori 26695
Rif2	CP003906	Rifampin-resistant strain selected from H. pylori 26695
Aklavik117	CP003483	Clinical isolate from Amerindian resident in Aklavik, Canada
Aklavik86	CP003476	Clinical isolate from Amerindian resident in Aklavik, Canada
OK113	AP012600	Collected in Okinawa Prefectural Chubu Hospital, Uruma, Okinawa, Japan
OK310	AP012601	Collected in Okinawa Prefectural Chubu Hospital, Uruma, Okinawa, Japan
UM032	CP005490	Isolated from a patient presenting with peptic ulcer disease
UM299	CP005491	Isolated from a patient presenting with peptic ulcer disease

UM037	CP005492	Isolated from a patient presenting with peptic ulcer disease
UM066	CP005493	Isolated from a patient presenting with peptic ulcer disease
UM298	CP006610	Isolated from a patient presenting with peptic ulcer disease
BM012A	CP006888	Collected from antral gastric biopsy specimens from volunteer at Sir Charles Gairdner Hospital, Nedlands, Western Australia
AM012S	CP006889	Collected from spouse of BM012A volunteer which was transmitted by the parental strain at Sir Charles Gairdner Hospital, Nedlands, Western Australia
oki102	CP006820	Isolated from a patient in Okinawa, Japan who had gastric atrophy
oki112	CP006821	Isolated from a patient in Okinawa, Japan who had gastric atrophy
oki128	CP006822	Isolated from a patient in Okinawa, Japan who had gastric atrophy
oki154	CP006823	Isolated from a patient in Okinawa, Japan who had duodenal ulcer
oki422	CP006824	Isolated from a patient in Okinawa, Japan who had gastric atrophy
oki673	CP006825	Isolated from a patient in Okinawa, Japan who had gastric ulcer
oki828	CP006826	Isolated from a patient in Okinawa, Japan who had duodenal ulcer
oki898	CP006827	Isolated from a patient in Okinawa, Japan who had duodenal ulcer
J166	CP007603	Clinical isolate strain used for experimental infection of rhesus monkeys
BM013A	CP007604	Collected from antral gastric biopsy specimens from the study participant during gastroendoscopy
BM012B	CP007605	Collected after 44 days of re-infection with BM012A to the participants
BM013B	CP007606	Collected after 20 days of re-infection with BM013A to the participant
Hp238	CP010013	Isolated from a Taiwanese patient with gastric MALT lymphoma
26695-1	CP010435	Sequenced as a wild type to compare with artificially induced drug resistant strains, Clarithromycin resistant strain
26695-IMET	CP010436	Metronidazole resistant strain
NY40	AP014523	Phage belong to a previously undescribed viral family, has been isolate
26695-1	AP013354	Sequenced as a wild type to compare with artificially induced drug resistant strains, Clarithromycin resistant strain
26695-1CL	AP013356	Sequenced as a wild type to compare with artificially induced drug resistant strains, Clarithromycin low resistant strain
26695-1CH	AP013355	Sequenced as a wild type to compare with artificially induced drug resistant strains, Clarithromycin high resistant strain
J99	CP011330	isolated in the USA in 1994 from a patient with a duodenal ulcer
Klebsiella pneumoniae		
HS11286	CP003200	Subspecies pneumoniae, isolated from a sputum specimen in 2011 at Huashan Hospital, Shanghai, China
NTUH-K2044	AP006725	Serotype K1, Subspecies pneumoniae, isolated from a patient with liver

		abscess and meningitis
MGH 78578	CP000647	Serotype K52, Subspecies pneumoniae, isolated from sputum of a 66 year-old man with pneumonia in 1994
KCTC 2242	CP002910	A 2,3-Butanediol producing, industrially important bacterium
KPNIH10	CP007727	Subspecies pneumoniae, isolated from rectal of 72 year old female patient no.10 related to the 2011 outbreak of carbapenem-resistant Klebsiella pneumoniae, patient died
KPNIH1	CP008827	Subspecies pneumoniae, isolated from groin of 43 year old female patient no.1 related to the 2011 outbreak of carbapenem-resistant Klebsiella pneumoniae, patient survive
1084	CP003785	Serotype K1, Subspecies pneumoniae, isolated from a diabetic patient with a bacteremic liver abscess at a referral medical center in central Taiwan between 2002 and 2004. Hypermucoviscosity-negative K1 clinical strain.
ATCC BAA-2146	CP006659	First Klebsiella strain isolated from patient with urinary tract infection in the U.S. to be reported as a "superbug" which is resistance to all β-lactam antibiotics except aztreonam
JM45	CP006656	Isolated from the blood of a 72-year-old male with cerebral infarction after colon carcinoma resection hospitalized in the intensive care unit, the Second Affiliated Hospital of ZheJiang University School of Medcine on April 7, 2010
CG43	CP006648	Serotype K2, isolated from liver abscess from Chang-Gung Memorial Hospital, virulent clinical isolate of K2 serotype which can synthesize aerobactin
Kp13	CP003999	Subspecies pneumoniae, isolated from blood culture of a patient admitted to the intensive care unit of a teaching hospital located in the city of Londrina, Southern Brazil
30684/NJST258_2	CP006918	Obtained in 2010 from patients with urinary tract infections at two separate healthcare institutions in New Jersey, multidrug-resistant.
30660/NJST258_1	CP006923	Obtained in 2010 from patients with urinary tract infections at two separate healthcare institutions in New Jersey, multidrug-resistant.
KPNIH27	CP007731	Subspecies pneumoniae, isolated from groin swabs of a patient of stem cell transplant in Jan 2012
KPNIH24	CP008797	Subspecies pneumoniae, isolated from throat/groin swabs of a patient of immunodeficiency in Jul 2012
KPR0928	CP008831	Subspecies pneumoniae, isolated from sputum of a patient of immunodeficiency in Oct 2012
PittNDM01	CP006798	Subspecies pneumoniae, isolated from urine of an inpatient at a hospital

		in Pittsburgh, Pennsylvania in March, 2013
blaNDM-1	CP009114	Isolated from clinical urine culture
ATCC 43816 KPPR1	CP009208	Subspecies pneumoniae, spontaneous rifampin-resistant isolate from ATCC 43816
PMK1	CP008929	Isolated from blood cultured from the first infected neonatal case
KPNIH33	CP009771	Subspecies pneumoniae, isolated from urine of patient
KPNIH32	CP009775	Subspecies pneumoniae, isolated from perirectal swabs of a patient with hematologic malignancy in Apr 2013
xH209	CP009461	Isolated from the blood of a patient in Hangzhou, Zhejiang, China, during tigecycline treatment
KPNIH29	CP009863	Subspecies pneumoniae, isolated from perirectal swabs of a patient of organ transplant in Nov 2013
KPNIH30	CP009872	Subspecies pneumoniae, isolated from perirectal swabs of a patient of hematologic malignancy in Nov 2013
KPNIH31	CP009876	Subspecies pneumoniae, isolated from urine of a patient of hematologic malignancy in Dec 2013
32192	CP010361	Isolated from excreted bodily substance of a patient
HK787	CP006738	Serotype K2
34618	CP010392	Isolated from bronchoalveolar lavage of a patient
1158	CP006722	Subspecies pneumoniae, serotype K2 strain from diabetic patient in Taiwan
Kp52.145	FO834906	Serotype K2, laboratory strain used to study Klebsiella pneumoniae pathogenesis
234-12	CP011313	Isolated from a blood culture in 2011 during an outbreak on a neonatal intensive care unit in Germany
SB3432	FO203501	Subspecies rhinoscleromatis, isolated in 2004 in the Avicenne hospital, Bobigny, France from a biopsy of the left nasal cavity of an 11-year old patient diagnosed with rhinoscleroma
CAV1392	CP011578	Isolated from sputum of patient
CAV1344	CP011624	Isolated from urine of urinary tract infection patient at Virginia, USA
CAV1596	CP011647	Isolated from perirectal from Virginia, USA
Listeria monocytogenes		
4b F2365	AE017262	Serotype 4b, isolated in 1985 in California, USA, during an outbreak of listeriosis among patients with AIDS which was caused by cheese product
HCC23	CP001175	Serotype 4a, isolated from channel catfish
08-5923	CP001604	Serotype 1/2a, isolated from a human blood specimen associated with

		the listeriosis outbreaks in Canada in 2008
Clip80459 serotype 4b	FM242711	Serotype 4b, isolated in a clinical outbreak of listeriosis in France affecting 42 persons
08-5578	CP001602	Serotype 1/2a, isolated from a human blood specimen associated with the listeriosis outbreaks in Canada in 2008
FSL R2-561	CP002003	Serotype 1/2c,
Finland 1998	CP002004	Serotype 3a, isolated from packaged dairy butter delivered to Tertiary Care Hospital by a Finnish dairy plant
J0161	CP002001	Serotype 1/2a, isolated from a case in a human listeriosis outbreak in 2000 linked to consumption of sliced turkey in the United States
10403S	CP002002	serotype 1/2a, streptomycin resistant isolate of strain 10403 the parental strain 10403 was first isolated from a human skin lesion obtained from Montana State University
J1816	CP006046	Serotype 4b, ECII strain involved in the 2002 U.S. outbreak associated with turkey deli meat isolated from the environment
J1-220	CP006047	Serotype 4b, ECIV strain involved in an outbreak associated with vegetables
L99	FM211688	Serotype 4a, isolated from food by Kampelmacher in 1950s in the Netherlands
M7	CP002816	Serotype 4a, isolated from cow's milk in Zhejiang province, China
07PF0776	CP003414	Serotype 4b, isolated from blood cultures from the patient by the Baystate Health Microbiology Laboratory
4b str. LL195	HF558398	Serotype 4b, isolated during the 1983–1987 listeriosis epidemic in Switzerland
R2-502	CP006594	Serotype1/2b, isolated from food in USA
C1-387	CP006591	Serotype 1/2a, isolated from turkey breast in 1999 at New York
J2-064	CP006592	Serotype 1/2b, carries one of the most common ribotypes found among food isolates, DUP-1052, and also is commonly associated with human disease
J2-031	CP006593	Serotype 1/2a, isolated from cow
J2-1091	CP006596	Serotype 1/2a, isolated from animal
N1-011A	CP006597	Serotype 1/2b, isolated from environmental sample, a non-outbreak sample
J1776	CP006598	Serotype 4b, ECII strain involved in the 2002 U.S. outbreak associated with turkey deli meat isolated from food
J1817	CP006599	Serotype 4b, ECII strain involved in the 2002 U.S. outbreak associated with turkey deli meat isolated from the environment
J1926	CP006600	Serotype 4b, ECII strain involved in the 2002 U.S. outbreak associated

		with turkey deli meat isolated from human sample
WSLC1001	CP007160	Serotype 1/2a, isolated from Germany
WSLC1042	CP007210	Serotype 4b, associated with outbreak and isolated from Germany
6179	HG813249	Serotype 1a, isolate from farmhouse cheese
EGD	HG421741	Serotype 1/2a, isolated from guinea pigs in 1926
NE dc2014	CP007492	Isolated from disease outbreak due to cheese from USA
R479a	HG813247	Serotype 1/2a, isolated from smoked salmon from Denmark and persisted from November 1996 to January 1999
CFSAN006122	CP007600	Isolated from disease outbreak due to cheese from USA
Lm60	CP009258	Serotype 1/2a, isolated from blood of a sporadic case of human listeriosis that shows enhanced cold stress tolerance in Switzerland
NTSN	CP009897	Serotype 4b, isolated from the brains of sheep in Jiangsu Province, China
IZSAM_Lm_hs2008	CP010346	Serotype 4b, isolated from cephalorachidian fluid of an immunocompetent adult male resident in Italy not belonging to any particular high-risk category who developed the disease and died
N2306	CP011004	Serotype 4b, isolated from a Swiss listeriosis outbreak in 2014 due to the consumption of contaminated ready-to-eat salads
CFSAN008100	CP011398	Isolated from green chili ingredient in USA
CFSAN007956	CP011397	-
La111	HE999704	Serotype 1/2a, isolated from a package of cold-smoked salmon in 1996
N53-1	HE999705	Serotype 1/2a, isolated from a Danish fish processing environment in 2002
EGD-e	AL591824	Serotype 1/2a, isolated from a rabbit tissue in Cambridge, England in 1924
serotype 7 str. SLCC2482	FR720325	Serotype 7, clinical isolates from human sample
L2676	CP007685	Serotype 1/2a, isolated from cantaloupe in US during 2011
L2624	CP007686	Serotype 1/2b, isolated from cantaloupe in US during 2011
L2625	CP007687	Serotype 1/2a, isolated from cantaloupe in US during 2011
L1846	CP007688	Serotype 1/2b, isolated from sporadic clinical isolate
L2074	CP007689	Serotype 1/2a, isolated from a human clinical isolate
Staphylococcus aureus		
04-02981	CP001844	MRSA
ED98	CP001781	Avian pathogen, isolated from a broiler chicken in Northern Ireland
RF122	AJ938182	Bovine pathogen, a common strain associated with mastitis in cattle
COL	CP000046	MRSA, resistant to several other antibiotics including penicillin and

		tetracycline
ED133	CP001996	Ovine pathogen
JH1	CP000736	VISA, the chronologically earliest strain was vancomycin sensitive
JH9	CP000703	VISA, exhibited increased vancomycin resistance
JKD6159	CP002114	MRSA
MRSA252	BX571856	MRSA, a hospital-acquired strain isolated in the United Kingdom
MSSA476	BX571857	MSSA, a hyper-virulent community acquired MSSA strain isolated in the United Kingdom
MW2	BA000033	MRSA, a community-acquired MRSA strain
Mu3	AP009324	VISA, comparative genome analysis
Mu50	BA000017	VISA, a MRSA strain with vancomycin resistance isolated in 1997
N315	BA000018	MRSA, a MRSA strain isolated in 1982 from a pharyngeal smear of a Japanese patient
NCTC 8325	CP000253	MSSA, the prototypical strain for most genetic research on *S. aureus*
ST398 S0385	AM990992	MRSA
TW20	FN433596	MRSA
USA300_FPR3757	CP000255	MRSA, strong association with unusually invasive disease, including severe septicemia, necrotizing pneumonia and necrotizing fasciitis
USA300_TCH1516	CP000730	MRSA, comparative analysis
JKD6008	CP002120	MRSA, resistant to methicillin and has intermediate sensitivity to vancomycin
Newman	AP009351	MRSA, comparative genome analysis
TCH60	CP002110	Reference genome for the Human Microbiome Project
55/2053	CP002388	Comparative genome analysis
T0131	CP002643	MRSA, isolated in 2006 from an 87-year-old patient in No.3 Central Hospital,Tianjin, China
M013	CP003166	MRSA, pvl-positive ST59:SCCmec V Strain, isolated in 2002 from a wound specimen of a pediatric outpatient during part of the Taiwan Surveillance of Antimicrobial Resistance surveillance project
LGA251	FR821779	Isolated from a bulk milk sample from a farm in southwest England in May, 2007
11819-97	CP003194	MRSA, Isolated from a skin infection in Denmark in 1997
VC40	CP003033	VRSA
ECT-R 2	FR714927	Multiresistant-MRSA, Human patient isolate with an unusual pattern of resistance against antibiotics
HO 5096 0412	HE681097	EMRSA, isolated from a fatal neonatal infection in Suffolk, UK in

		February 2005
08BA02176	CP003808	MRSA, obtained in 2008 from a human postoperative surgical site infection
16035	HE579065	MRSA, collected between 2001 and 2008 at the tertiary care hospital of Lausanne
16125	HE579067	MRSA, collected between 2001 and 2008 at the tertiary care hospital of Lausanne
18583	HE579073	MRSA, collected between 2001 and 2008 at the tertiary care hospital of Lausanne
CA-347	CP006044	MRSA, isolated from a bacteremia infection in 2005 in California
Bmb9393	CP005288	MRSA, isolated in 1993 from a case of nosocomial bloodstream infection in Rio de Janeiro, Brazil
6850	CP006706	MSSA, derived from a patient with a complicated S. aureus bacteremia, associated with osteomyelitis and septic arthritis
CN1	CP003979	MRSA, obtained from the pus of a necrotizing fasciitis infection in an 80-year old patient in the Seoul area of South Korea
SA957	CP003603	MRSA, representative strains of Taiwan clone
SA40	CP003604	MRSA, representative strains of Asian-Pacific clone
Z172	CP006838	MRSA, isolated in 2010 from a blood specimen of an elderly intensive care unit patient during part of the Taiwan Surveillance of Antimicrobial Resistance project
USA300-ISMMS1	CP007176	MRSA, isolated from liver transplant recipient that despite transmission from a deceased donor with MRSA endocarditis and bacteremia
502A	CP007454	Bacterial Interference Strain, isolated in 1963 from a nurse in a newborn nursery who was caring for a cluster of 40 neonates who also became colonized with this strain
NRS100	CP007539	MRSA, laboratory strain with tetracycline resistant
H-EMRSA-15	CP007659	MRSA, isolated from wound
UA-S391_USA300	CP007690	A prolific biofilm formers isolated from abscess or wound
XN108	CP007447	MRSA, isolated from a steam-burned patient with a wound infection
SA268	CP006630	MRSA, isolated from both the blood and the sputum of a young patient with severe sepsis and acute respiratory failure in March of 2012
2395 USA500	CP007499	MRSA, isolated from a wound infection which shown hypervirulent in a murine model of systemic infection and highly cytotoxic towards primary human neutrophils
ATCC 25923	CP009361	a standard laboratory testing control strain for susceptibility testing to antibiotics and as a quality control strain for commercial products

Gv69	CP009681	Isolated from a hospitalized patient presented with wound infection in Teresina, PI, Brazil in 1996
FORC_001	CP009554	Isolated from one of the real sample which caused food-borne outbreaks in South Korea
29b_MRSA	CP010295	MRSA, isolate was propagated in ATCC medium 18, tryptic soy agar, and has been utilized as a model *S. aureus* strain
31b_MRSA	CP010296	MRSA, isolate was propagated in ATCC medium 18, tryptic soy agar, and has been utilized as a model *S. aureus* strain
33b	CP010297	MRSA, isolate was propagated in ATCC medium 18, tryptic soy agar, and has been utilized as a model *S. aureus* strain
26b_MRSA	CP010298	MRSA, isolate was propagated in ATCC medium 18, tryptic soy agar, and has been utilized as a model *S. aureus* strain
25b_MRSA	CP010299	MRSA, isolate was propagated in ATCC medium 18, tryptic soy agar, and has been utilized as a model *S. aureus* strain
27b_MRSA	CP010300	MRSA, isolate was propagated in ATCC medium 18, tryptic soy agar, and has been utilized as a model *S. aureus* strain
ST772-MRSA-V strain DAR4145	CP010526	MRSA, blood sample isolation from Mumbai, India
ILRI Eymole1/1	LN626917	Isolated from nasal swab of camel from Kenya
10388	HE579059	MRSA, collected between 2001 and 2008 at the tertiary care hospital of Lausanne
10497	HE579061	MRSA, collected between 2001 and 2008 at the tertiary care hospital of Lausanne
15532	HE579063	MRSA, collected between 2001 and 2008 at the tertiary care hospital of Lausanne
18341	HE579069	MRSA, collected between 2001 and 2008 at the tertiary care hospital of Lausanne
18412	HE579071	MRSA, collected between 2001 and 2008 at the tertiary care hospital of Lausanne
FCFHV36	CP011147	MRSA, isolated from a vertebral biopsy sample from a patient diagnosed with community-acquired osyteomyelitis and under medical care in the hospital in the state of Santa Catarina, Brazil
71193	CP003045	MSSA, isolated in 2004 from human
M1	HF937103	MRSA, clinical isolate from outbreak in Copenhagen, Denmark, that started in 2003
M121	CP007670	MRSA, isolated from nasal swab from healthy volunteer at Columbia
CA15	CP007674	Isolated from blood with bacteremia
Salmonella		

enterica		
D23580	FN424405	serovar Typhimurium
SL483	CP001138	serovar Agona, causes gastroenteritis in humans and is also pathogenic to swine and other food animals
SC-B67	AE017220	serovar Choleraesuis, an extremely invasive serovar that is increasingly becoming resistant to multiple antibiotics such as fluoroquinolones
CT_02021853	CP001144	serovar Dublin, This is a bovine-adapted serovar that is genetically related to S. Enteritidis that lives in the bovine intestinal tract and can causes disease in humans
P125109	AM933172	serovar Enteriditis, a virulent phage type 4 strain
287/91	AM933173	serovar Gallinarum, the causative agent of Fowl typhoid, a severe systemic disease of poultry
SL476	CP001120	serovar Heidelberg, a multidrug resistant strain
SL254	CP001113	serovar Newport, an multidrug resistant strain from one of two distinct lineages of the Newport serovar
AKU_12601	FM200053	serovar Paratyphi A, a clinical isolate collected from a child with paratyphoid fever in Karachi, Pakistan, 2004
ATCC 9150	CP000026	serovar Paratyphi A, a narrow host range and causes a typhoid-like illness in humans
SPB7	CP000886	serovar Paratyphi B, susceptible to antibiotics, classified as serovar Paratyphi B
RKS4594	CP000857	serovar Paratyphi C, harbors a large pathogenicity island, SPI7, with genes coding for the virulence antigen
CVM19633	CP001127	serovar Schwarzengrund, predominant cause of Salmonellosis in Southeast Asia
CT18	AL513382	serovar Typhi, a multidrug resistant strain of Salmonella typhi
Ty2	AE014613	serovar Typhi, no multidrug resistance plasmids and has been used for vaccine development
14028S	CP001363	serovar Typhimurium, substrain of strain LT2
LT2	AE006468	serovar Typhimurium, isolated in the 1940s by Lilleengen
SL1344	FQ312003	serovar Typhimurium
ST4/74	CP002487	serovar Typhimurium, isolated from a calf with salmonellosis in the United Kingdom
507440-20	CP007530	serovar Montevideo, causes Pepper Salami Outbreak
UK-1	CP002614	serovar Typhimurium
RKS5078	CP003047	Serovar Gallinarum biovar Pullorum, chicken-adapted pullorum agent closely related to another chicken pathogen, *Salmonella enterica*

		serovar Gallinarum
P-stx-12	CP003278	serovar Typhi, clinical isolate obtained from a typhoid carrier in India
798	CP003386	serovar Typhimurium, causes persistent infections in pigs and can spread to other animals and cause food-borne disease in humans
B182	CP003416	serovar Heidelberg, isolated from bovine feces in France
CVM 21550	CP010283	serovar Newport, isolated from swine
CVM 22513	CP010281	serovar Newport, isolated from cattle
CVM 21538	CP010282	serovar Newport, isolated from chicken
CVM 22425	CP010279	serovar Newport, isolated from cattle
CVM N18486	CP009561	serovar Newport, isolated from ground turkey
CVM N1543	CP010284	serovar Newport, isolated from ground beef
CVM 22462	CP010280	serovar Newport, isolated from canine
77-1427	CP007598	serovar Enteritidis, clinical isolate
T000240	AP011957	serovar Typhimurium, isolated from human gastroenteritis in Japan in 2000
CDC_2010K_0968	CP007528	serovar Enteritidis, clinical isolate
18569	CP011394	serovar Enteritidis, poultry isolate from Mexico
CFSAN001992	CP004027	serovar Javiana, common food-borne pathogen and is often associated with fresh-cut produce
Ty21a	CP002099	serovar Typhi, an important vaccine for controlling typhoid fever and serves as an oral vector for delivering heterologous antigens
CFSAN002069	CP005390	serovar Heidelberg, isolated from an unopened chicken sample collected from the patient's home at Washington State
41578	CP004086	serovar Heidelberg, clinical isolate
CFSAN002050	CP006055	serovar Cubana, isolated from fresh alfalfa sprouts in Arizona in 2012
CFSAN001921	CP006048	serovar Typhimurium, var. 5, isolated from chicken breast meat
08-1736	CP006602	serovar Typhimurium, isolated from pig samples in Spain in 1997
CFSAN000189	CP006053	serovar Bareilly, isolated from raw frozen shrimp
USMARC-S3124.1	CP006631	serovar Newport, isolated from cattle
S06004	CP006575	serovar Pullorum, a nalidixic acid-resistant clinical isolate obtained in 2006 from chickens in Jiangsu China
CDC1983-67	CP003786	serovar Gallinarum biovar Pullorum
RM6836	CP006717	serovar Thompson, isolated from lettuce in 2002
460004 2-1	CP011259	serovar Agona, isolated from unsweetened puffed-rice cereal in Minnesota in 2008
ATCC 8391	CP011396	serovar Thompson, isolated from fresh bagged lettuce

TXSC_TXSC08-19	CP007505	serovar Tennessee, isolated from fishmeal at Texas in US
ATCC BAA-1592	CP007531	serovar Anatum, isolated from tomatoes in Pennsylvania
0014	CP007534	serovar Abony
CFSAN001080	CP007533	serovar Bredeney, isolated from Marjoram
ATCC 35640	CP007532	serovar Abaetetuba, isolated from Creek Water, Argentina, Zaiman
DT2	HG326213	serovar Typhimurium, isolated from *Columba livia*
DT104	HF937208	serovar Typhimurium, widely disseminated zoonotic pathogen with multidrug-resistant
24249	CP006876	serovar Agona, isolated from ready to eat meat from a factory at Ireland
CFSAN002064	CP005995	serovar Heidelberg, isolated from stool of outbreak case patient which had consume a specific brand of chicken
Durban	CP007507	serovar Enteritidis, isolated from a patient with salmonellosis and typhoid fever
EC20121175	CP007269	serovar Enteritidis, isolated from chick paper at Manitoba, Canada
EC20121179	CP007272	serovar Enteritidis, isolated from quail at Quebec, Canada
SA20093266	CP007274	serovar Enteritidis, isolated from chicken feces at Ontario, Canada
EC20110223	CP007266	serovar Enteritidis, isolated from human sample during mung bean outbreak at Ontario, Canada
EC20120005	CP007267	serovar Enteritidis, isolated from broiler at Ontario, Canada
EC20110361	CP007263	serovar Enteritidis, isolated from sporadic human sample (2009) at Alberta, Canada
EC20110360	CP007258	serovar Enteritidis, isolated from sporadic human sample (2004) at Alberta, Canada
EC20110359	CP007259	serovar Enteritidis, isolated from sporadic human sample (2004) at Alberta, Canada
EC20110358	CP007260	serovar Enteritidis, isolated from sporadic human sample (2009) at Alberta, Canada
EC20110357	CP007261	serovar Enteritidis, isolated from sporadic human sample (2003) at Alberta, Canada
EC20110356	CP007262	serovar Enteritidis, isolated from human outbreak sample (2009) at Alberta, Canada
EC20090135	CP007320	serovar Enteritidis, isolated from chicken in Ontario, Canada
EC20090193	CP007321	serovar Enteritidis, isolated from chicken in Ontario, Canada
EC20090332	CP007322	serovar Enteritidis, isolated from chicken in Ontario, Canada
EC20120916	CP007332	serovar Enteritidis, isolated from chicken in Ontario, Canada
EC20100103	CP007420	serovar Enteritidis, isolated from chicken in Ontario, Canada

EC20090884	CP007421	serovar Enteritidis, isolated from chicken in Ontario, Canada
EC20090531	CP007422	serovar Enteritidis, isolated from chicken in Ontario, Canada
EC20110354	CP007175	serovar Enteritidis, isolated from hospitalized patient sample during outbreak (2010) at Alberta, Canada
EC20120008	CP007245	serovar Enteritidis, isolated from reptile in Ontario, Canada
EC20110221	CP007247	serovar Enteritidis, isolated from human sample during mung bean outbreak in Ontario, Canada
EC20090698	CP007248	serovar Enteritidis, isolated from abattoir Sporadic chicken sample in Alberta, Canada
EC20090641	CP007249	serovar Enteritidis, isolated from abattoir Sporadic chicken sample in Alberta, Canada
EC20110355	CP007250	serovar Enteritidis, isolated from food –breast outbreak sample in Alberta, Canada
EC20110353	CP007251	serovar Enteritidis, isolated from food –pork dumpling outbreak sample at Alberta, Canada
EC20111175	CP007252	serovar Enteritidis, isolated from environmental- chicken fluff in Quebec, Canada
EC20111174	CP007253	serovar Enteritidis, isolated from environmental- chicken fluff in Quebec, Canada
EC20111095	CP007254	serovar Enteritidis, isolated from human outbreak sample in Quebec, Canada in 2011
EC20120009	CP007438	serovar Enteritidis, isolated from rodent sample in Ontario, Canada
EC20120929	CP007463	serovar Enteritidis, isolated from chicken in Ontario, Canada
SA20084644	CP007466	serovar Enteritidis, isolated from avian in Ontario, Canada
138736	CP007581	serovar Typhimurium, stool sample from patient with gastroenteritis in Israel
VNP20009	CP007804	serovar Typhimurium, a derivative of 14028S and was selected after UV and chemical mutagenesis for tumor-colonizing and growth-inhibiting properties
C500	CP007639	serovar Choleraesuis, isolated from chicken intestinal in Yangzhou, China
ATCC 13311	CP009102	serovar Typhimurium, genomic DNA isolated from *Salmonella enterica* subsp. Serovar Typhimurium strain NCTC 74
OLF-SE7-100819	CP009089	serovar Enteritidis
OLF-SE8-1021710	CP009090	serovar Enteritidis
OLF-SE9-10012	CP009091	serovar Enteritidis

OLF-SE10-1005 2	CP009092	serovar Enteritidis
OLF-SE11-1005 8	CP009093	serovar Enteritidis
OLF-SE3-98983 -4	CP009085	serovar Enteritidis
OLF-SE2-98984 -6	CP009084	serovar Enteritidis
OLF-SE4-0317- 8	CP009086	serovar Enteritidis
OLF-SE5-1104- 2	CP009087	serovar Enteritidis
OLF-SE1-1019- 1	CP009083	serovar Enteritidis
OLF-SE6-00219 -16	CP009088	serovar Enteritidis
SEJ	CP008928	serovar Enteritidis,
CMCC 50973	CP009049	serovar Paratyphi A, isolated from human in Jiangsu, China
CMCC 50503	CP009559	serovar Paratyphi A, isolated from human in Jiangsu, China
L-3553	AP014565	serovar Typhimurium, isolated from cattle in Hokkaido, Japan since 2004
EC20100134	CP007359	serovar Enteritidis, isolated from chicken in Ontario, Canada
EC20100130	CP007358	serovar Enteritidis, isolated from chicken in Ontario, Canada
CDC 06-0532	CP007211	serovar Anatum, isolated from human stool sample
ARS-USMARC-1927	CP007216	serovar Newport, isolated from livestock fecal swab
USDA-ARS-US MARC-1903	CP007222	serovar Montevideo, isolated from ground beef
USDA-ARS-US MARC-1899	CP007235	serovar Typhimurium, isolated from ground beef
SINFA	LN649235	serovar Infantis, isolated from healthy chicken
USDA-ARS-US MARC-1175	CP007483	serovar Anatum, isolated from ground beef
CDC 2011K-0870	CP007523	serovar Typhimurium, isolated from human stool sample
USDA-ARS-US MARC-1921	CP007540	serovar Montevideo, isolated from human stool sample

CDC 2010K-2159	CP007559	serovar Newport, isolated from human stool sample
USDA-ARS-USMARC-1735	CP007584	serovar Anatum, isolated from bovine pre-evisceration carcass of Bos taurus
FORC_015	CP011365	serovar Typhimurium, isolated from human blood culture in Korea
YU39	CP011428	Serovar Typhimurium, isolated from a male 8 year old suffering from hepatomegaly and severe thrombocytopenia
Streptococcus pyogenes		
M1 GAS	AE004092	Serovar M1, a strict human pathogen that was isolated from a patient with a wound infection
MGAS10270	CP000260	Serovar M2, for comparative genome analysis
MGAS10394	CP000003	Serovar M6, a Serovar M6 isolate cultured from a child with pharyngitis in private elementary school in Pennsilvania
MGAS10750	CP000262	Serovar M4, for comparative genome analysis
MGAS2096	CP000261	Serovar M12, for comparative genome analysis
MGAS315	AE014074	Serovar M3, a Serovar M3 strain
MGAS6180	CP000056	Serovar M28, isolated from an invasive disease in Texas in 1998
MGAS8232	AE009949	Serovar M18, associated with acute rheumatic fever outbreaks in the USA
MGAS9429	CP000259	Serovar M12, for comparative genome analysis
NZ131	CP000829	Serovar M49, isolated from a patient with acute glomerulonephritis
SSI-1	BA000034	Serovar M3, isolated from a toxic-shock patient in Japan and associated with severe invasive infections, necrotizing fasciitis, and death
Manfredo	AM295007	Serovar M5, associated with acute rheumatic fever isolated from a patient in the 1950's in Chicago
Alab49	CP003068	Serovar M53, recovered from an impetigo lesion in Alabama in 1986
MGAS15252	CP003116	Serovar M59, group A streptococcus, caused an epidemic of severe invasive human infections, spread west to east across Canada over a 3-year period (2006 to 2008)
MGAS1882	CP003121	Serovar M59, strain deposited in the BEI Resources as NR-33708
HKU QMH11M0907901	AFRY01000001	Serovar M12, bacteremic strain from a patient with scarlet fever in Hong Kong
A20	CP003901	Serovar M1, isolated from a blood sample from a patient with necrotizing fasciitis in Tainan Provincial Hospital
M1 476	AP012491	Serovar M1, isolated from a patient with streptococcal toxic shock syndrome during pregnancy

HSC5	CP006366	Serovar M14, a robust producer of the cysteine protease SpeB and is capable of producing infection in several different animal models
STAB901	CP007024	Serovar M44, group A streptococcus, Tetracycline-resistant, isolated from the blood of a patient with endometritis
STAB902	CP007041	Serovar M3, isolated from a superficial pyodermatitis
ATCC 19615	CP008926	Group A nonmotile β-hemolytic clinical isolate is used for quality control in a variety of commercially available tests
M23ND	CP008695	Serovar M23, isolated from an invasive human infection
7F7	CP007240	Serovar M83, isolated among homeless persons in Brittany, France
HKU360	CP009612	Serovar M12, isolated from Scarlet fever outbreak between 2005-2011 in Hong Kong and mainland China
1E1	CP007241	Serovar M44, isolated from human sample in Brittany, France
AP1	CP007537	Serovar M1, isolated from a blood bacteremia infected patient
JRS4	CP011414	Serovar M6, a streptomycin-resistant derivative strain from the Rockefeller University Lancefield collection with rheumatic fever
D471	CP011415	Serovar M6, a strain from the Rockefeller University Lancefield collection with rheumatic fever
NGAS743	CP007560	Isolated from human sample
NGAS596	CP007561	Isolated from human sample
NGAS327	CP007562	Isolated from human sample
MGAS5005	CP000017	Serovar M1, GroupA streptococcus, isolated from a clinical specimen of cerebrospinal fluid in Toronto, Canada in 1996
5448	CP008776	Serovar M1, clinical strain
M28PF1	CP011535	Serovar M28, isolated from a patient with postpartum endometritis
